# Supplementary material for: Genomic Safe Harbor Expression of PAX7 for the Generation of Engraftable Myogenic Progenitors
Source: Stem Cell Reports. 2020 Dec 3;16(1):10–9. doi: 10.1016/j.stemcr.2020.11.001 (PMC7815936; doi:10.1016/j.stemcr.2020.11.001)
Supplement: Document S1. Supplemental Experimental Procedures, Figures S1–S3, and Tables S1–S3 [file mmc1.pdf]

Stem Cell Reports, Volume *16*

## Supplemental Information

# Genomic Safe Harbor Expression of PAX7 for the Generation of Engraftable Myogenic Progenitors

**Hyunkee Kim, Sridhar Selvaraj, James Kiley, Karim Azzag, Bayardo I. Garay, and Rita C.R. Perlingeiro**

## Supplemental Figure 1

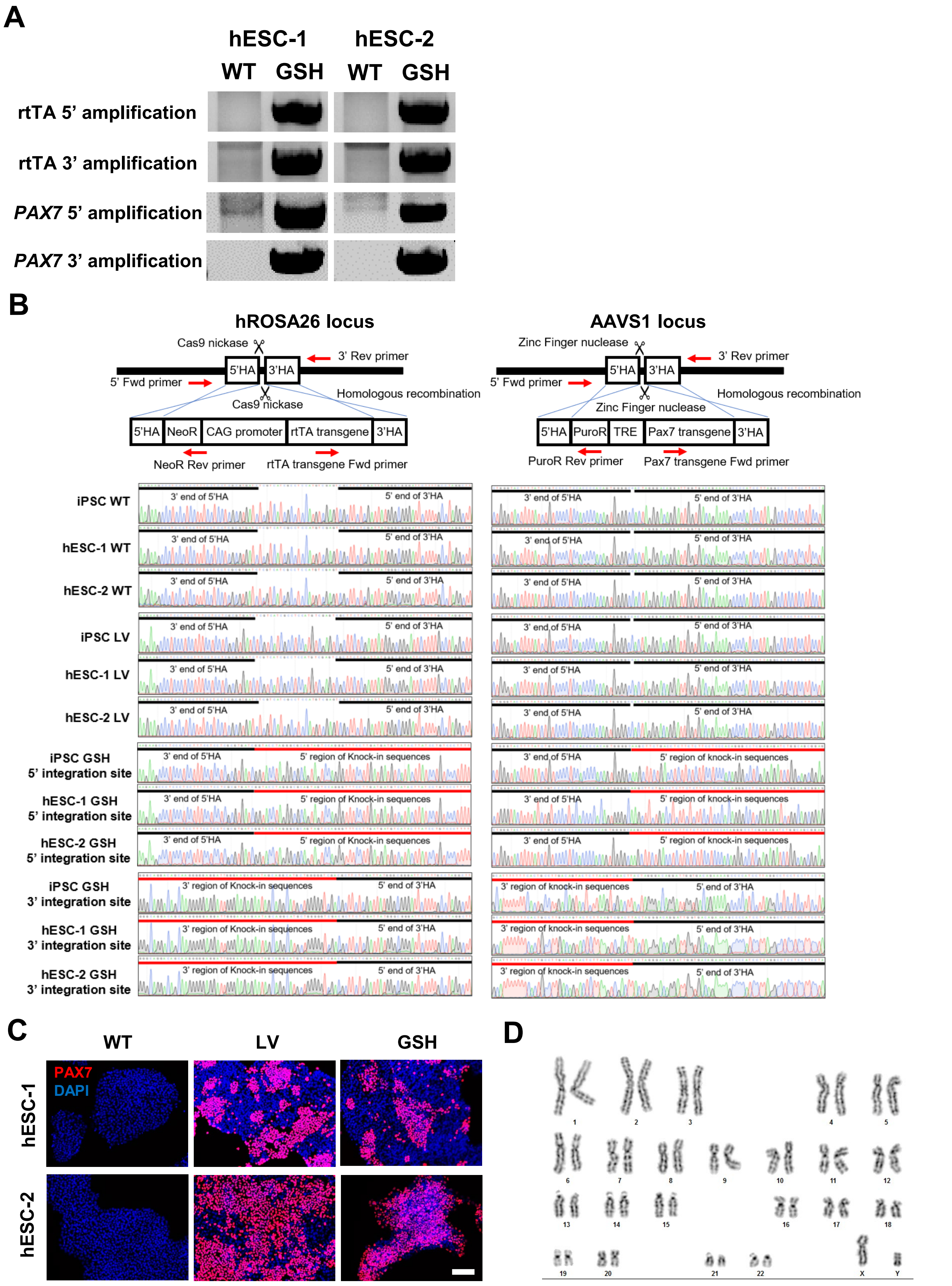

# Supplemental Figure 2

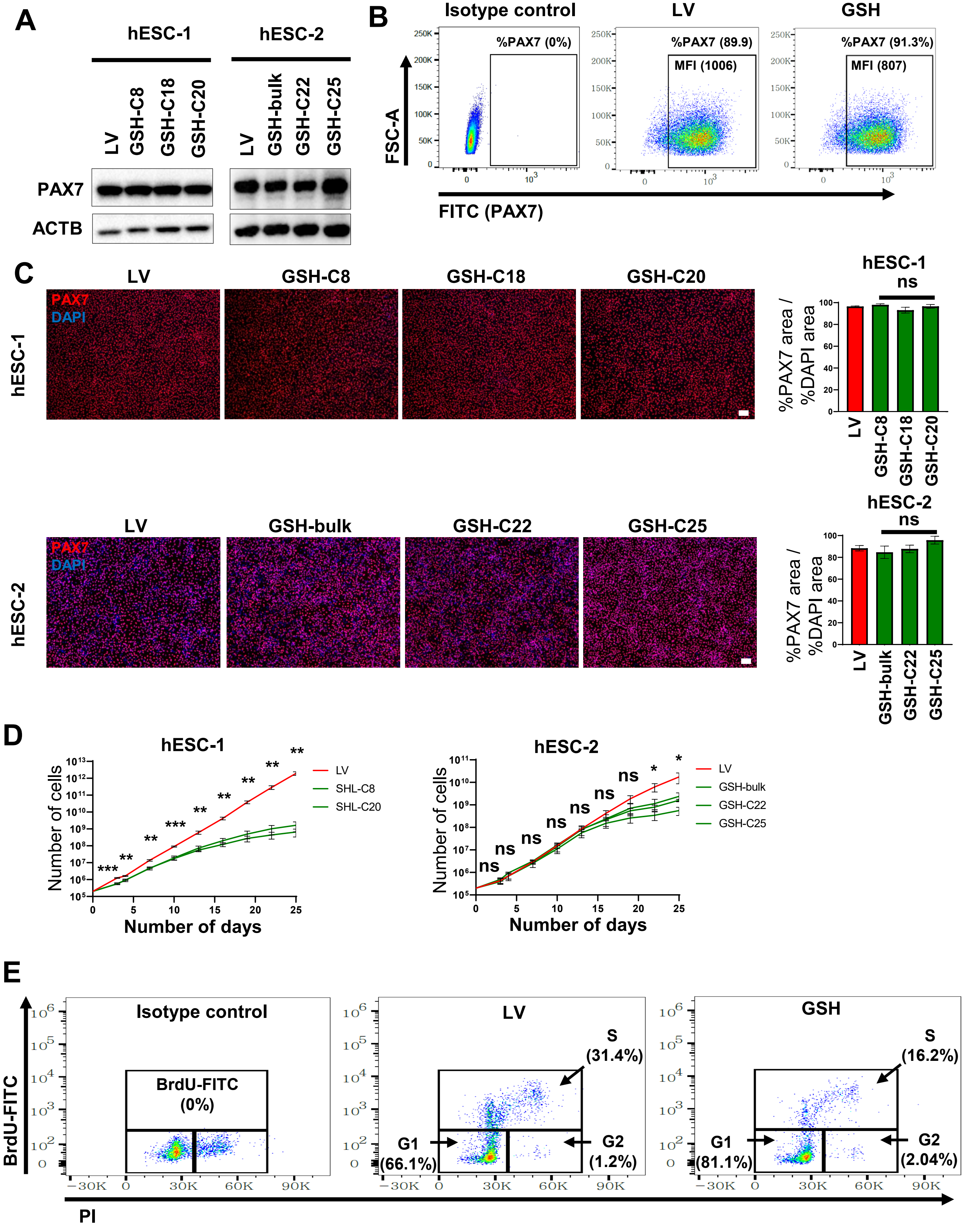

# Supplemental Figure 3

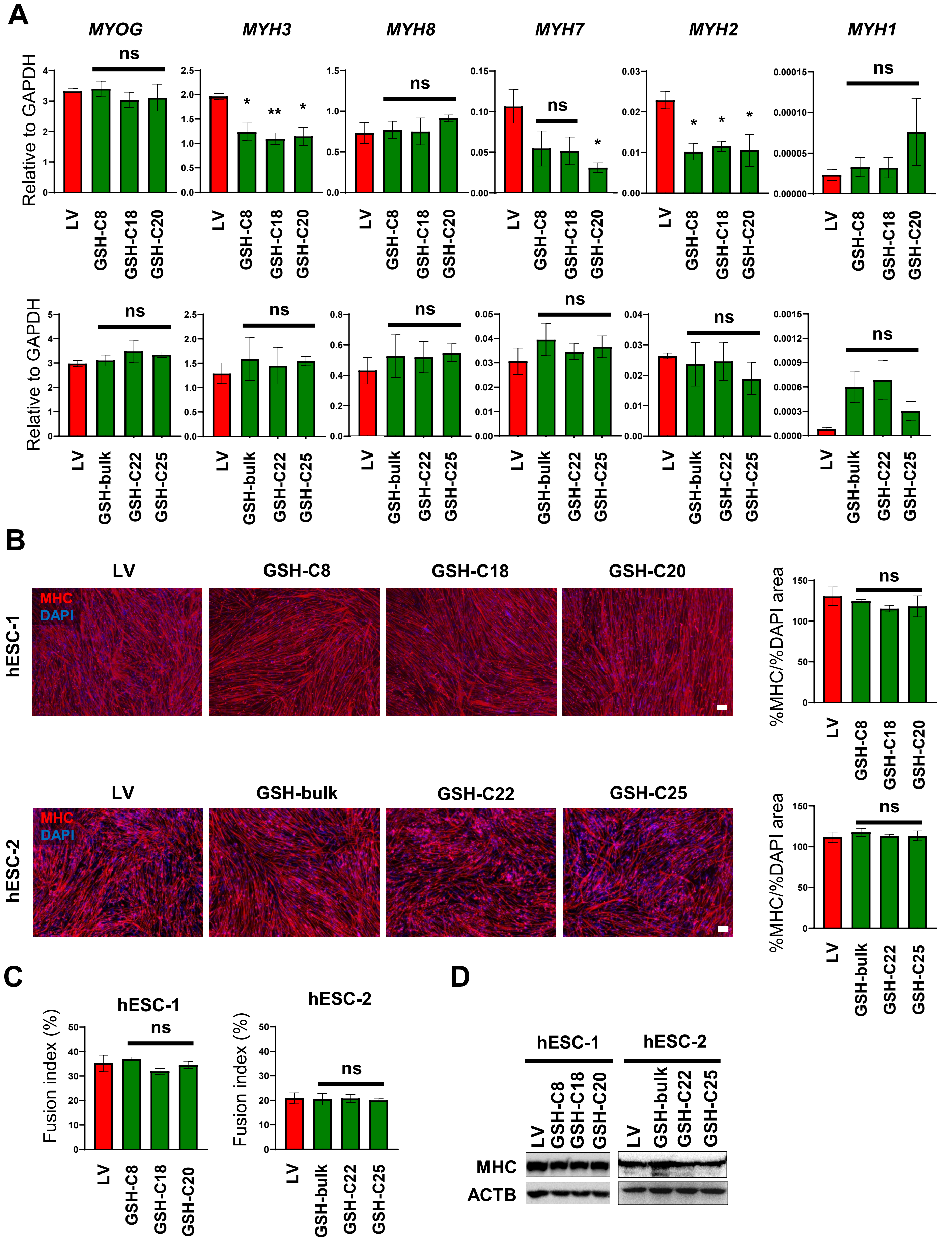

## SUPPLEMENTAL FIGURE LEGENDS

**Figure S1. Related to Figure 1: Confirmation of *PAX7* and rtTA insertion into respective AAVS1 and ROSA26 loci in additional hPSC lines.**
(A) PCR shows integration analysis of *PAX7* and rtTA transgenes into the AAVS1 and ROSA26 loci in hESC-1 and hESC-2 lines. Primers were designed outside of the 5'- and 3'-homology arms and within the transgenes. WT: wild-type parental hESC; GSH: genomic safe harbor targeted hESC.
(B) Sequencing results of the rtTA and *PAX7* transgene knock-in in the ROSA26 and AAVS1 loci. Primers amplifying knock-in specific sequences were used to obtain the amplicons for sequencing. Black lines indicate intact sequences whereas red lines indicate knock-in sequences.
(C) Immunofluorescence staining shows PAX7 protein expression upon 1 day of dox induction in hESC-1 and hESC-2 lines. Of note, this staining was performed in the bulk population prior to clonal selection. PAX7 staining is shown in red. DAPI in blue stains nuclei. WT and LV hESC-1/hESC-2 were used as negative and positive controls, respectively. Scale bar, 100 μm.
(D) Cytogenetic analyses show normal karyotype for representative GSH iPSC line (TC1133).

**Figure S2. Related to Figure 2: Characterization of GSH PAX7$^+$ myogenic progenitors derived from the bulk and selected clones from targeted hESC-1 and hESC-2 lines.**
(A) Western blot for PAX7. β-Actin was used as a loading control.
(B) Representative FACS plots for PAX7 in LV and GSH myogenic progenitors. Percentage for PAX7 and the mean fluorescence intensity (MFI) are indicated on respective plots. Isotype antibody served as negative control.
(C) Left panels show representative images of immunofluorescence staining for PAX7 (red) in GSH and LV CD54$^+$SDC2$^+$ myogenic progenitors derived from hESC-1 (upper panels) and hESC-2 (lower panels) lines. DAPI (blue) stains nuclei. Scale bar, 100 μm. Graph bars (right panels) show ratio of % PAX7-stained area to % DAPI area in GSH and LV myogenic progenitors. Data are shown as the mean ± S.E.M. of three independent replicates.
(D) Growth curves of GSH and LV CD54$^+$SDC2$^+$ myogenic progenitors derived from hESC-1 (left) and hESC-2 (right) lines. Cells were counted every 3 days. Data are shown as the mean ± S.E.M. of three independent replicates. *$p<0.05$, **$p<0.01$, ***$p<0.001$
(E) Representative FACS plots for cell cycle analysis. Isotype antibody served as negative control.

**Figure S3. Related to Figure 3: Terminal differentiation of GSH hESC-derived myogenic progenitors into myotubes.**
(A) Graph bars show gene expression of *MYOGENIN (MYOG)* and *MYOSIN-HEAVY-CHAIN* (*MYH*) isoforms in GSH and LV hESC-1 (upper) and hESC-2 (lower) myotubes. Data are shown as the mean ± S.E.M. of three independent replicates. *$p<0.05$, **$p<0.01$
(B) Left panels show representative images of immunofluorescence staining for MHC (red) in GSH and LV hESC-1 (upper) and hESC-2 (lower) myotubes. DAPI (blue) stains nuclei. Scale bar, 100 μm. Graph bar (right panel) shows ratio of % MHC-stained area to % DAPI area in GSH and LV myotubes derived from hESC-1 and hESC-2 lines. Data are shown as the mean ± S.E.M. of three independent replicates.
(C) Bar graph shows the quantification of fusion index of hESC-derived LV and GSH myotubes. Data are shown as the mean ± S.E.M. of three independent replicates.
(D) Western blot for MHC. β-Actin was used as a loading control.

## SUPPLEMENTAL TABLES

### Table S1. Primers for genotyping PCR

| Locus | PCR types | Binding site of primers | Sequences |
|---|---|---|---|
| AAVS1 | 5' region integration | Endogenous genome (5')-Forward | TAGCCACTCTGTGCTGACCACTCTG |
| | | PuroR (Vector)- Reverse | GAAGAGTTCTTGCAGCTCGGTGAC |
| | 3' region integration | PAX7 (Vector)- Forward | CTGTCTCCTCAGGTGATGAGCATCTTG |
| | | Endogenous genome (3')- Reverse | ACTGAGGGTTTCAGTGCTAAAACTAGGCTGT |
| | Locus PCR | Endogenous genome (5')- Forward | TAGCCACTCTGTGCTGACCACTCTG |
| | | Endogenous genome (3')- Forward | AGCTCTTCTCTGTTCAGCCCTAAGAATC |
| hROSA26 | 5' region integration | Endogenous genome (5')- Forward | ACATAATGTTTGTTACGTTGGGAGGGAAAG |
| | | NeoR (Vector)- Reverse | ATTGCATCAGCCATGATGGATACTT |
| | 3' region integration | rtTA (Vector)- Forward | GAAACTCGCTCAAAAGCTGGG |
| | | Endogenous genome (3')- Reverse | ACAGTACAAGCCAGTAATGGAG |
| | Locus PCR | Endogenous genome (5')- Forward | GTCTCTTTTCTGTCGGACCCTTACCTTGTC |
| | | Endogenous genome (3')- Forward | ATCAGAGTATACTAGGAGCTCAGGAGTACAAGA |

### Table S2. SYBR green qRT-PCR primers

| Target | Primers for SYBR green | Primer sequences |
|---|---|---|
| Endogenous PAX7 | 5'-UTR forward primer | TGTGTGTGGAGGGGAGGGAGAA |
| | PAX7 Exon2 reverse primer | GCTGATTGACCCGGCCTTGG |

### Table S3. Southern blot primers for probes

| Southern blot probes | Primers for probe | Primer sequences |
|---|---|---|
| rtTA probe | rtTA forward primer | CCGCTGTGCTCTCCTCTCACATCG |
| | rtTA reverse primer | TCAGAAGTGGGGGCATAGAATCGG |
| PAX7 probe | PAX7 forward primer | CAAGTTCGGGAAGAAAGAGGA |
| | PAX7 reverse primer | GTATATGTCTGGGTAGTGGGTCCT |

## SUPPLEMENTAL EXPERIMENTAL PROCEDURES

**Generation of PAX7$^+$rtTA$^+$ GSH PSC lines.**
hROSA26 gRNA/Cas9n and AAVS1 ZFN expression plasmids, as well as pR26_CAG-rtTA and pAAVS1_TRE targeting plasmids were kindly provided by Dr. Mark Kotter from the University Cambridge, UK (Pawlowski et al., 2017). We used SpeI/EcoRI sites to clone PAX7 (Darabi et al., 2012) into the pAAVS1_TRE targeting plasmid. hROSA26 gRNA/Cas9n expression plasmids and pR26_CAG-rtTA targeting plasmid were delivered to hPSCs by nucleofection (Lonza), as previously described (Pawlowski et al., 2017). Following G418 treatment (50 µg/mL for 10 days), neomycin resistant cells were selected. Subsequently, AAVS1 ZFN expression plasmids and pAAVS1_TRE-PAX7 targeting plasmid were delivered through nucleofection using the same procedure. Following puromycin treatment (500 ng/mL) for 10 days, resistant clones were selected and verified by knock-in specific PCR, sequencing, and protein expression. Sequences of PCR primers can be found in Table S1.

**hPSC maintenance and myogenic differentiation**
The following PSC lines were used: TC1133 iPS cells, and human ES cell lines H1 (hESC-1) and H9 (hESC-2). Cells were maintained in mTeSR1 medium (Stem Cell Technologies) on Matrigel-coated plates. PSCs were dissociated with Accutase (Innovative Cell Technologies) and passaged once they reached 90% confluency. For differentiation, PSCs (LV and GSH) were dissociated into single cells using Accutase, and differentiated into embryoid bodies (EBs) in the presence of EB myogenic medium, which consists of IMDM containing 4% fetal bovine serum, 11% KnockOut Serum Replacement (Gibco), 1% insulin-transferrin selenium (Gibco), 1% penicillin/streptomycin (Gibco), 1% Glutamax (Gibco), and 50 µg/mL ascorbic acid (Sigma). Doxycycline (dox; Sigma-Aldrich) induction (1 µg/ml) started on day 5 of EB differentiation. On day 8, medium was further supplemented with 5 ng/ml of human basic fibroblast growth factor (bFGF; PeproTech). On day 12, EBs were dissociated with 0.25% trypsin-EDTA, and plated as monolayer. On day 15, monolayer cultures were rinsed with PBS, and dissociated with Cellstripper (Corning) for fluorescence-activated cell sorting (FACS), as described below. CD54+SDC2 sorted cells were expanded in the EB myogenic medium described above with 1 µg/mL dox (Sigma) and 5ng/mL human basic FGF (PeproTech). For myotube differentiation, we used >95% confluent myogenic progenitors. These were rinsed with PBS to remove dox, and cultured in low serum conditions, as described previously (Darabi et al., 2012).

**FACS analysis and sorting**

To purify myogenic progenitors, cells were first stained with the primary antibodies biotin-conjugated CD54 (Thermo Fisher) and APC-conjugated SYNDECAN2 (SDC2; R&D systems) for 20 min, as previously described (Magli et al., 2017). Streptavidin PE-Cy7 (Thermo Fisher) was used for secondary staining. Cells were washed with PBS and then resuspended in PBS supplemented with 10% FBS. For PAX7 staining, cells were dissociated with 0.25% trypsin-EDTA, fixed with 4% PFA at room temperature for 10 min, and washed twice with PBS. Cell permeabilization was performed using FACS buffer containing 0.2% Tween, 0.5% Saponin, 4% FBS, and 0.4% 0.5M EDTA on a shaker for 30 min at room temperature. Next, cells were incubated with the PAX7 antibody (mouse; PAX7-s; DSHB) in FACS buffer without 0.2% Tween 20 at room temperature for 45 min. Cells were washed twice with PBS and incubated with an anti-mouse secondary antibody (anti-mouse Alexa Fluor 488) in FACS buffer without 0.2% Tween 20 for 45min. Cells were washed twice with PBS, and resuspended in FACS buffer containing 4% FBS, and 0.4% 0.5M EDTA. Samples were analyzed and sorted using a FACSAria II (BD Biosciences) and data were analyzed with FlowJo software.

**Transplantation studies**

Animal experiments were carried out according to protocols approved by the University of Minnesota Institutional Animal Care and Use Committee. Both TA muscles of 6-8 week-old NOD-scid IL2Rgnull (NSG; Jackson) or 10-12 week-old NOD-scid IL2Rgnull mdx4cv (NSG-mdx4cv) (Arpke et al., 2013) or 10-12 week-old NOD-scid IL2Rgnull C3KO (NSG-C3KO) (Selvaraj et al., 2019a) mice were pre-injured with 15 µl of cardiotoxin 10 µM (Latoxan). One day after cardiotoxin, TA muscles were injected with $10^6$ myogenic progenitors. GSH myogenic progenitors were injected in one TA while the contralateral TA received LV myogenic progenitors, as internal control. CTX-injured TA muscles injected with PBS served as negative. TA muscles were collected for immunofluorescence analysis 8-9 weeks later.

**Quantitative RT-PCR (qPCR)**

Cells were lysed using Trizol reagent (Thermo Fisher) and RNA was extracted using purelink RNA mini kit (Thermo Fisher) with on-column DNAse treatment following manufacturer's instructions. RNA concentration was quantified using Nanodrop. For quantitative RT-PCR analysis, reverse transcription was performed using Superscript Vilo cDNA synthesis kit (Thermo Fisher) as per manufacturer's instruction. qPCR was performed using taqman probes (Applied Biosystems). For each qPCR reaction in 384-well plate, cDNA amount corresponding to 10 ng of total RNA, 0.5 µl of taqman probe or 0.2 µM of forward and reverse SYBR green primers and 5 µl of 2X master mix was utilized. qPCR was performed using QuantStudio 6 Flex Real-Time PCR System and the $C_t$ values were determined. $C_t$ value for gene of interest was normalized to that of the housekeeping control using the 2^-delta $C_t$ calculation and values compared between the GSH and LV groups. Following are the taqman probes used in this study, *T* (Hs01084475_g1), *FOXC2* (Hs00270951_s1), *MEOX1* (Hs00244943_m1), *TCF15* (Hs00231821_m1), *PAX3* (Hs00992437_m1), *PAX7* (Hs00242962_m1), *PAX7* variant2 (Hs01557428_mH), *MYH1* (Hs00428600_m1), *MYH2* (Hs00430042_m1), *MYH3* (Hs01074230_m1), *MYH7* (Hs01110632_m1), *MYH8* (Hs00267293_m1), *MYOG* (Hs01072232_m1), and *GAPDH* (Hs99999905_m1). SYBR green primers are listed in Table S2.

**Immunofluorescence staining**

Cells were fixed at room temperature with 4% PFA for 30 min, followed by 15 min permeabilization with 0.3% Triton X-100 in PBS. Samples were then blocked with 3% BSA in PBS for 1 hr. We used the following primary and secondary antibodies: PAX7 (mouse; PAX7-s; DSHB), MHC (mouse; MF-20-s; DSHB), human DYSTROPHIN (mouse; MANDYS106(2C6)-s; DSHB), human LAMIN A/C (rabbit; ab108595; Abcam), LAMININ α2 (rat; sc-59854; Santa Cruz), and fluorophore-conjugated secondary antibodies (anti-mouse Alexa Fluor 555 (A-21426), anti-rabbit Alexa Fluor 488 (A-11008), and anti-rat Alexa Fluor 647 (A-21247); Thermo Fisher). Samples were incubated in primary antibody diluted in 3% BSA at 4°C overnight. Next day, samples were washed with PBS three times and incubated with secondary antibody and DAPI at room temperature for 1 hr. After incubation, samples were washed with PBS three times and stored at 4°C in dark until imaging. For tissue, muscle samples were cryosectioned at 14 µm thickness on glass slides. Sectioned tissues were then rehydrated in PBS for 5 min, fixated with 4% PFA for 10 min, and stained as described above. After the final wash, slides were mounted with coverslips by using ProLong Gold Antifade Mountant with DAPI (Thermo Fisher), and stored at 4°C in the dark until imaging. Samples were imaged using upright (Zeiss) and confocal (NikonNiE C2) microscopy. Quantification was performed using the Fiji software.

**Western blot analysis**

Cells were lysed in lysis buffer containing 20 mM Tris-HCl (Sigma), 0.1 mM EDTA (Sigma), 1 mM DDT (Sigma), 28 µM E64 (Sigma), 20 µg/mL soybean trypsin inhibitor (Sigma), and 2 mM phenylmethylsulfonyl fluoride (PMSF; Santa Cruz), and 1X Laemmli sample buffer. Cell scraper was used to collect lysate and the lysate was

boiled at 95°C for 5-10 min. Total protein concentration in lysate was measured by using a Bradford assay. 7.5% SDS-PAGE gels were used for electrophoresis and migrated proteins were transferred to immobilon-FL polyvinylidene fluoride (PVDF) membrane (Millipore). Transferred membrane was blocked in 5% dry milk (RPI) in PBST (PBS and 0.1% Tween 20) for 1 hr at room temperature. Blocked membrane was then incubated with primary antibody diluted in 5% BSA in PBST and incubated at 4°C overnight. Next day, membrane was washed three times in PBST and incubated with horseradish peroxidase (HRP)-conjugated secondary antibody for 1 hr at room temperature. Membrane was then washed three times with PBST and then Supersignal West chemiluminescent substrate (Thermo Fisher) was applied to image in Bio-Rad ChemiDoc MP imaging system. Antibodies used include TetR (mouse; 631131; Clontech), PAX7 (mouse; PAX7-s; DSHB), MHC (mouse; MF-20-s; DSHB), ACTB (mouse; sc-47778; Santa Cruz), and TUBB (mouse; sc-5274; Santa Cruz).

**Southern blot analysis**
Genomic DNA was extracted using the Purelink ™ Genomic DNA mini kit as per manufacturer's instructions. Genomic DNA was digested overnight at 37°C with BstXI restriction enzymes for detection of the rtTA transgene detection, and with XmnI for detection of *PAX7*. Following electrophoresis in 1% agarose gel, DNA was transferred to positively charge nylon membrane and probed with digoxigenin (DIG)-labeled rtTA and *PAX7* probes. DIG-labeled probes were synthesized by using PCR DIG Probe Synthesis Kit (Roche) and detection was performed by using DIG-High Prime DNA Labeling and Detection Starter Kit II (Roche). Primer sequences utilized for generating probes are listed in Table S3.

**Cell cycle**
For assessment of cell cycle we used the previously reported protocol (Zhu, 2012). Briefly, cells were treated with 30 µM BrdU (Sigma) for 8 hr, and following trypsinization, fixed and permeabilized with 70% ice-cold EtOH overnight at 4°C. For DNA denaturation, we used 2 N HCl with 0.5% Triton-X-100. Cells were washed first with 0.1 M sodium tetraborate (pH 8.5) in 1X PBS solution, and then with 1% BSA in 1X PBS. Cells were incubated with a FITC-conjugated BrdU antibody (mouse; 364104; BioLegend) for 1hr at room temperature in the dark, washed with 1% BSA in 1X PBS, resuspended in 1X PBS containing 10 µg/mL RNase A and 20 µg/mL propidium iodide, and incubated at room temperature for 30 min in the dark. Samples were analyzed immediately or stored at 4°C in the dark until FACS analysis. FlowJo software was used for data analysis.

## SUPPLEMENTAL REFERENCES

Zhu, H. (2012). Cell proliferation assay by flow cytometry (BrdU and PI staining). Bio-101 2, e198.
