# Supplementary material for: Genomic Safe Harbor Expression of PAX7 for the Generation of Engraftable Myogenic Progenitors
Source: Stem Cell Reports. 2020 Dec 3;16(1):10–9. doi: 10.1016/j.stemcr.2020.11.001 (PMC7815936; doi:10.1016/j.stemcr.2020.11.001)
Supplement: Document S2. Article plus Supplemental Information [file mmc2.pdf]

Stem Cell Reports
Report
OPEN ACCESS

Genomic Safe Harbor Expression of PAX7 for the Generation of Engraftable Myogenic Progenitors

Hyunkee Kim,[1,2] Sridhar Selvaraj,[1,2] James Kiley,[1] Karim Azzag,[1] Bayardo I. Garay,[1] and Rita C.R. Perlingeiro[1,2,3,*]
[1]Lillehei Heart Institute, Department of Medicine, University of Minnesota, 4-128 CCRB, 2231 6th Street SE, Minneapolis, MN 55455, USA
[2]Department of Genetic, Cell Biology, and Development, University of Minnesota, Minneapolis, MN, USA
[3]Stem Cell Institute, University of Minnesota, Minneapolis, MN, USA
*Correspondence: perli032@umn.edu
https://doi.org/10.1016/j.stemcr.2020.11.001

SUMMARY

Inducible expression of PAX7 in differentiating pluripotent stem cells (PSCs) allows massively scalable generation of human myogenic progenitors, which upon transplantation into dystrophic muscles give rise to donor-derived myofibers and satellite cells. Therefore, PSC-derived PAX7$^+$ myogenic progenitors represent an attractive therapeutic approach to promote muscle regeneration. Work to date has used lentiviral vectors (LVs) that randomly integrate inducible *PAX7* transgenes. Here, we investigated whether equivalent induction of the myogenic program could be achieved by targeting the *PAX7* transgene into genomic safe harbor (GSH) sites. Across multiple PSC lines, we find that this approach consistently generates expandable myogenic progenitors *in vitro*, although scalability of expansion is moderately reduced compared with the LV approach. Importantly, transplantation of GSH-targeted myogenic progenitors produces robust engraftment, comparable with LV counterparts. These findings provide proof of concept for the use of GSH targeting as a potential alternative approach to generate therapeutic PSC-derived myogenic progenitors for clinical applications.

INTRODUCTION

Muscular dystrophies (MDs) encompass more than 40 different genetic disorders characterized by progressive skeletal muscle degeneration and weakness, which in severe disorders result in paralysis and death (Emery, 2002). Despite significant progress in understanding disease pathogenesis and in developing therapeutic strategies, there is still no cure for MDs.

Much current effort is focused on the development of adeno-associated virus (AAV)-mediated gene therapy approaches, which have advanced to the clinical trial stage for the treatment of Duchenne MD (Bowles et al., 2012; Mendell et al., 2010), among a few others (Crudele and Chamberlain, 2019). However, safety and efficacy remain major issues (Goswami et al., 2019). One significant challenge is the immune response against the AAV vector, allowing only a single-dose delivery, which may not be sufficient to provide long-term therapeutic benefit.

Another potential therapeutic strategy consists of replacing diseased muscle by transplanting healthy muscle stem cells. Major caveats here include the accessibility to sufficient number of stem cells from a given donor without damaging the muscle, and the reduction of engraftment potential following their *ex vivo* expansion (Mendell et al., 1995; Montarras et al., 2005). An alternative approach is to utilize human pluripotent stem cells (hPSCs) to generate transplantable muscle stem cells. PSCs are appealing for therapeutic application as they can repeatedly produce large amounts of differentiated tissue, providing an unlimited source of cells for therapy. However, a critical aspect for translation is their controlled lineage-specific differentiation, since transplantation of contaminating undifferentiated PSCs can lead to tissue abnormalities or even give rise to teratomas. There are several strategies for the derivation of myogenic cells from PSCs, which include the use of *PAX7* or *MYOD* transgene overexpression (Albini et al., 2013; Darabi et al., 2012; Goudenege et al., 2012; Tedesco et al., 2012; Young et al., 2016) and transgene-free methods (Barberi et al., 2007; Chal et al., 2016; Shelton et al., 2014; Xi et al., 2017). Each strategy has unique advantages and disadvantages. Transgene-free methods are attractive, since these do not involve genetic manipulation; however, there are significant limitations in generating large numbers of therapeutically relevant myogenic cells. Moreover, these cultures possess a higher probability of carrying non-myogenic cells, including undifferentiated PSCs (Kim et al., 2017a), an issue that can be minimized by the use of efficient purification protocols (Hicks et al., 2018; Wu et al., 2018). Overexpression of transcription factors associated with the myogenic hierarchy assures the muscle identity of *in vitro* generated cells. In the case of PAX7, we have documented the generation of large numbers of early myogenic progenitors that upon transplantation into dystrophic mice results in myofiber and satellite cell engraftment (Darabi et al., 2012), suggesting potential for future therapeutic application.

Because lentiviral vectors (LVs) deliver transgenes through random integration, they convey some risk of insertional mutagenesis. To bypass this, we previously investigated the use of the integration-free minicircle vector to generate

This is an open access article under the CC BY license (http://creativecommons.org/licenses/by/4.0/).

PSC-derived PAX7+ myogenic progenitors (Kim et al., 2017b). Although the minicircle strategy showed efficient *in vitro* differentiation of hPSCs into myotubes, due to the transient nature of the minicircle vector, multiple transfections were required to maintain PAX7 expression. Furthermore, this inefficiency in maintaining high levels of PAX7 expression resulted in heterogeneous cultures, which failed to contribute to muscle engraftment (Kim et al., 2017b).

Targeted integration of a *PAX7* transgene into a genomic safe harbor (GSH) locus, such as the AAVS1, represents an attractive alternative approach. This locus is widely recognized as a potential ideal site for transgene insertion for gene therapy applications (Papapetrou and Schambach, 2016; Smith et al., 2008). Dual GSH targeting, an improved inducible GSH system that utilizes two different GSH loci, has been recently developed (Pawlowski et al., 2017). Advantages of this system include bypassing the promoter interference of two transgenes (Baron and Bujard, 2000) and providing the flexibility of transgene design as a result of targeting two different GSH loci.

Here, we used the dual GSH targeting approach to generate a highly expandable population of PAX7+ myogenic progenitors from hPSCs that upon transplantation contribute to *in vivo* muscle regeneration. These findings demonstrate the feasibility for the potential future use of transgene targeting into GSH loci as an approach for generating therapeutic hPSC-derived PAX7+ myogenic progenitors.

RESULTS

Targeting of PAX7 and rtTA Transgenes into GSH Sites

As outlined in Figure 1A, we inserted the doxycycline (dox)-inducible *PAX7* transgene into the AAVS1 locus and the rtTA (tetracycline-responsive transcriptional activator) transgene into the ROSA26 locus using the dual GSH targeting approach (Pawlowski et al., 2017). We confirmed integration of *PAX7* and rtTA transgenes into respective GSH loci by knockin-specific PCR (Figures 1B and S1A) and sequencing (Figure S1B). We confirmed protein expression of both PAX7 and rtTA by western blot and immunofluorescence staining following 1 day of dox treatment (Figures 1C, 1D, and S1C). LV hPSC counterparts for each respective line served as positive controls. We confirmed that the integration of *PAX7* and rtTA into both GSH sites did not affect the karyotype of genome engineered PSCs (Figure S1D). Next, we performed Southern blots to verify targeted integration. Our results show specific integration of rtTA and *PAX7* transgenes at the ROSA26 and AAVS1 loci, respectively (yellow arrow), while this was not the case for the LV approach (Figure 1E). Since the *PAX7* probe can hybridize into endogenous exons 4 and 5, two probed bands are detected for *PAX7* in all samples (black arrows, Figure 1E).

Generation of GSH Myogenic Progenitors

GSH and LV iPAX7 hPSCs were differentiated into embryoid bodies (EBs), and dox treatment began on day 5, as previously described (Selvaraj et al., 2019b). Day-4 EBs showed similar expression of the early mesodermal marker *T* (Figure 2A), and no differences were observed for the somitic mesodermal genes *FOXC2*, *PAX3*, and *TCF15* in day-6 EBs (Figure 2A). We detected lower expression levels of *PAX7* and *MEOX1* in GSH EBs compared with LVs (Figure 2A). This noticeably reduced expression of *PAX7* 1 day after the start of dox induction may be due to the lower copy number of the *PAX7* transgene in the GSH approach (Figure 2A), which then likely directly affects the expression levels of the PAX7 target, *MEOX1*. On day 15, PAX7+ myogenic progenitors were purified based on the expression of CD54 and Syndecan2 (SDC2), as described by Magli et al. (2017). We observed no differences in the frequency of CD54+SDC2+ myogenic progenitors between GSH and LV approaches (Figure 2B). Next, we assessed PAX7 expression in expanding myogenic progenitors. qRT-PCR results show that LV myogenic progenitors display higher expression levels of total and exogenous (transgene) *PAX7* than GSH myogenic progenitors (Figure 2C). No differences were found in the expression levels of endogenous *PAX7*, which were low in both LV and GSH myogenic progenitors (Figure 2C), indicating that PAX7 expression in these cells is virtually exclusively from the *PAX7* transgene. Comparable PAX7 protein expression was observed in GSH and LV myogenic progenitors by western blot (Figure S2A), fluorescence-activated cell sorting (FACS) (Figures 2D and S2B), and immunofluorescence (Figures 2F and S2C) staining. The only difference noted was the intensity of PAX7 expression in the FACS plots, as evidenced by the geometric mean value, which was found to be higher in LV myogenic progenitors (Figure 2E).

One of the major characteristics of the LV conditional expression system is the remarkable expansion potential. Therefore, we evaluated whether GSH-generated PAX7+ myogenic progenitors would have comparable expansion capacity compared with LV counterparts. Overall, GSH myogenic progenitors generated from all three PSC lines showed significant expansion potential (Figures 2G and S2D), but the LV-generated myogenic progenitors displayed a clear advantage in scalability, in particular in two of the three PSC lines evaluated (hiPSC-TC1133 and hESC-1). This resulted in greater than an order of magnitude more myogenic progenitor cells at the end of the 3-week expansion period. We then performed cell-cycle analyses by combining bromodeoxyuridine incorporation with propidium iodide staining (Figures 2H and S2E). Our results show that a lower proportion of GSH myogenic progenitors were in S phase compared with LV myogenic progenitors ($p < 0.05$), whereas the opposite was true for the G_1 phase

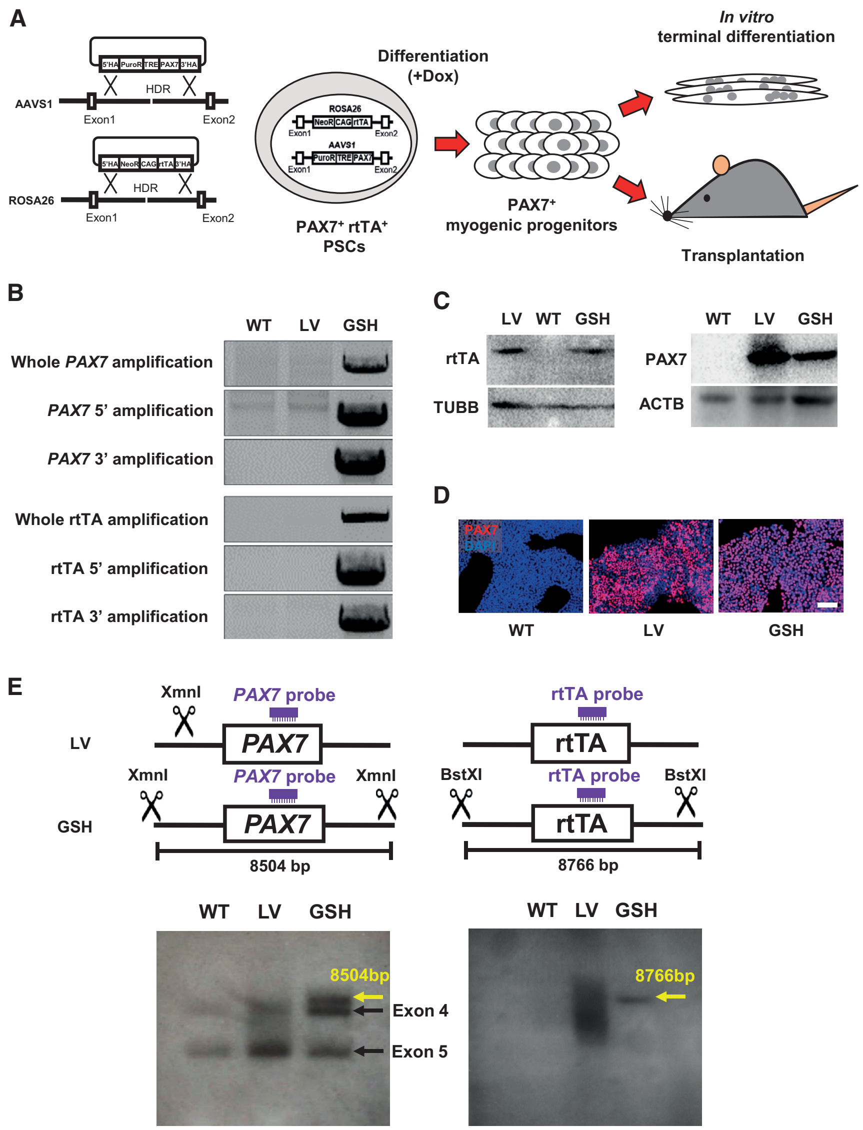

Figure 1. Generation of PAX7-Induced PSCs by GSH Loci Targeting

(A) Schematic representation of performed studies.

(B) PCR shows integration analysis of *PAX7* and rtTA transgenes into the AAVS1 and ROSA26 loci. Primers were designed outside of the 5′ and 3′ homology arms and within the transgenes. WT, wild-type parental induced pluripotent stem cell (iPSC); LV, lentivirus transduced iPSC; GSH, genomic safe harbor targeted iPSC.

(C) Western blots show PAX7 and rtTA expression. WT and LV iPSCs were used as negative and positive controls, respectively. β-Tubulin (TUBB) and β-actin (ACTB) were used as loading controls.

(D) Immunofluorescence staining shows PAX7 expression (in red) upon 1 day of dox induction. DAPI in blue stains nuclei. WT and LV iPSCs were used as negative and positive controls, respectively. Scale bar, 100 μm.

(E) Southern blot shows integration of *PAX7* and rtTA transgenes. *PAX7* and rtTA probes and digestion sites of XmnI and BstXI enzymes are indicated in the diagram (upper panel). Yellow arrows indicate specific integration of *PAX7* and rtTA transgenes in respective GSHs. Black arrows indicate endogenous *PAX7* at exons 4 and 5. WT iPSCs served as negative control.

(more of GSH progenitors were in G_1 phase), confirming that GSH myogenic progenitors proliferate more slowly than LV myogenic progenitors. This was reflected in the doubling time, as the average ± SEM for LV and GSH myogenic progenitors (all three PSC lines) was 30.8 ± 3.1 and 47.2 ± 1.6 h, respectively. Thus, taken together these results indicate that LV myogenic progenitors were endowed with greater expansion potential than GSH myogenic progenitors due to the attribution of increased proliferation.

In Vitro Terminal Differentiation of GSH PAX7$^+$ Myogenic Progenitors

Next, we differentiated GSH- and LV-generated myogenic progenitors into myotubes. We observed comparable gene-expression levels for *MYOG* and several *MYH* isoforms between the two approaches (Figures 3A and S3A). Quantification of myosin heavy chain (MHC) protein levels revealed no differences between GSH and LV myotubes (Figures 3B, 3D, S3B, and S3D). Moreover, comparable fusion indices were observed (Figures 3C and S3C). These data indicate that GSH-generated myogenic progenitors can efficiently give rise to MHC$^+$ myotubes *in vitro*.

Transplantation of GSH-Generated PAX7$^+$ Myogenic Progenitors

To determine the *in vivo* regenerative potential of GSH-generated PAX7$^+$ myogenic progenitors, we transplanted these cells into cardiotoxin pre-injured tibialis anterior (TA) muscles of NSG mice. Assessment of injected muscles 2 months later revealed the presence of donor-derived

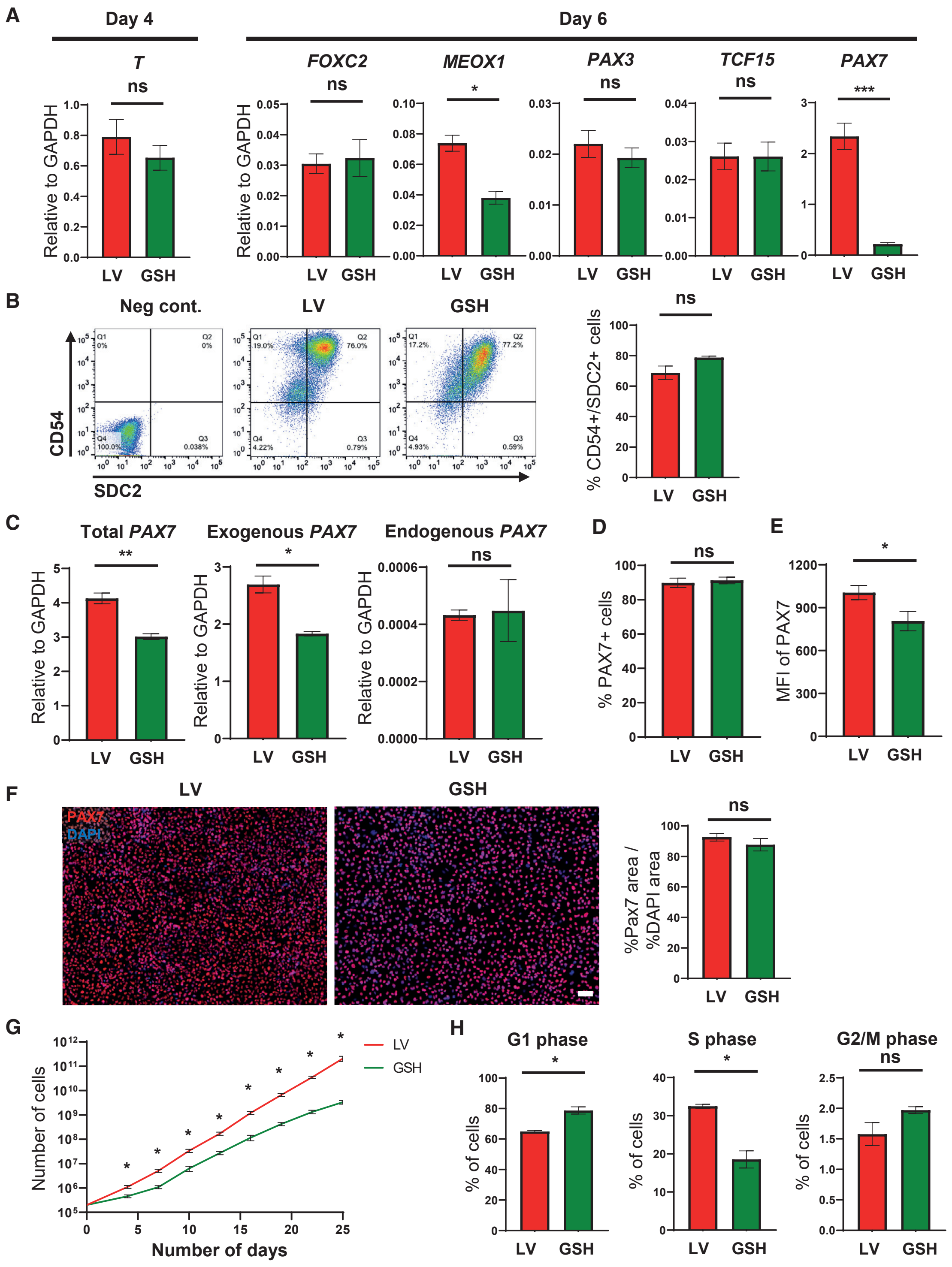

Figure 2. Characterization of GSH PAX7$^+$ Myogenic Progenitors

(A) Bar graphs show expression analysis of paraxial mesoderm (*T*) at day 4, and somite (*FOXC2*, *MEOX1*, *PAX3*, and *TCF15*) genes as well as *PAX7* at day 6 of EB differentiation for GSH and LV PAX7-induced iPSCs. Data are shown as mean ± SEM of six independent replicates. *p < 0.05, ***p < 0.001. ns, not significant.

(B) Representative FACS plots show the frequency of CD54$^+$SDC2$^+$ myogenic progenitors in GSH and LV PAX7-induced iPSC-derived EBs at day 15 of differentiation (left), and respective quantification (right). Data are shown as the mean ± SEM of three independent replicates. ns, not significant.

(legend continued on next page)

myofibers, as shown by immunofluorescence staining using human DYSTROPHIN and human LAMIN A/C specific antibodies, thus confirming engraftment in recipients transplanted with GSH-generated PAX7^{+} myogenic progenitors (Figure 4A). No staining was detected in PBS-injected controls (Figure 4A, left). Importantly, quantification of human donor-derived DYSTROPHIN^{+} fibers showed that GSH- and LV-generated PAX7^{+} myogenic progenitors have similar engraftment potential (Figure 4B).

Since satellite cell engraftment is critical to ensure long-term muscle regeneration, we investigated the ability of transplanted myogenic progenitors to give rise to satellite cells. For this, we stained muscle sections with antibodies to the satellite cell marker PAX7, human LAMIN A/C, and laminin-α2 (LM). Immunofluorescence staining showed that GSH-generated PAX7^{+} myogenic progenitors were able to seed the satellite cell compartment of transplanted muscles (Figure 4C, lower panel). Quantification revealed approximately 3% human donor contribution to the satellite cell pool, and no differences between the two approaches (Figure 4D). This level of engraftment is in agreement with our previous xenograft studies (Darabi et al., 2012). Of note, much higher numbers of donor-derived satellite cells were detected in our mouse-to-mouse transplantation studies in the presence or absence of pre-conditioning (Azzag et al., 2020; Incitti et al., 2019).

The *in vivo* engraftment potential of GSH-generated PAX7^{+} myogenic progenitors was also validated in immunodeficient mouse models of DMD (Arpke et al., 2013) and limb girdle muscular dystrophy type 2A (Selvaraj et al., 2019a). Comparable engraftment for LV and GSH myogenic progenitors (Figures 4E and 4F) was observed in both models, as shown by the presence of donor-derived myofibers expressing human DYSTROPHIN and human LAMIN A/C.

DISCUSSION

Conditional expression of PAX7 allows for the derivation of therapeutically relevant human myogenic progenitors (Darabi et al., 2012; Kim et al., 2017a; Magli et al., 2017; Selvaraj et al., 2019a). However, these studies have used an LV-based approach to deliver the *PAX7* transgene, which requires consideration of risks of integrational mutagenesis. On the other hand, GSH sites allow for the insertion of exogenous DNA material with robust expression at specific sites within the host genome, reducing the risk of insertional mutations (Papapetrou and Schambach, 2016).

Several studies have reported the targeting of GSH sites in patient-specific induced PSCs to insert wild-type sequences for the correction of monogenic diseases, including X-linked chronic granulomatous disease and hemophilia B (De Ravin et al., 2016; Lyu et al., 2018; Zou et al., 2011). To date, only Pawlowski et al. (2017) have applied this strategy to induce the *in vitro* differentiation of hPSCs into specific lineages, but these studies were solely *in vitro*. Here we report the targeting of GSH sites to generate engraftable hPSC-derived PAX7^{+} myogenic progenitors, as shown by their ability to contribute to myofibers and satellite cells *in vivo*, in a manner equivalent to their LV-generated counterparts.

One aspect in which the two approaches differed was the *in vitro* expansion potential, as GSH myogenic progenitors presented slower proliferation rates than LV-generated myogenic progenitors (doubling time of 47.2 ± 1.6 and 30.8 ± 3.1 h, respectively), which concurred with our cell-cycle assessment. One potential hypothesis for this difference may be the presence of fewer copies of the *PAX7* transgene in the GSH compared with the LV approach. Our results show increased *PAX7* gene expression (total and exogenous) in LV myogenic progenitors (Figure 2C), but this did not result in significant differences in PAX7 protein expression (Figures 2D, 2F, and S2A–S2C), although we noted higher PAX7 intensity by FACS in LV myogenic progenitors (Figure 2E). Collins et al. (2009) have shown that constitutive expression of PAX7 in satellite cells and C2C12 myoblasts leads to enhanced proliferative rate, and this also applied to fibroblasts upon PAX7 ectopic expression. We have reported similar results in differentiating mouse and human PSCs (Darabi et al., 2011; Kim et al., 2017b). Therefore, these studies suggest

(C) Bar graphs show expression analysis of total, exogenous, and endogenous *PAX7* in LV and GSH myogenic progenitors. Data are shown as mean ± S.E.M. of three independent replicates. $^{*}p < 0.05$, $^{**}p < 0.01$. ns, not significant.
(D and E) FACS for PAX7. Bar graphs show the quantification of PAX7 expression (D) and the mean fluorescence intensity (MFI), also known as geometric mean (E) in LV and GSH myogenic progenitors. Data are shown as the mean ± SEM of three independent biological replicates performed in triplicate. $^{*}p < 0.05$. ns, not significant.
(F) Representative images show immunofluorescence staining for PAX7 (red) in GSH and LV CD54^{+}SDC2^{+} myogenic progenitors (left panels). DAPI (blue) stains nuclei. Scale bar, 100 μm. Right panel shows the ratio of percent PAX7-stained area to percent DAPI area in GSH and LV myogenic progenitors. Data are shown as the mean ± SEM of three independent replicates. ns, not significant.
(G) Growth curves of GSH and LV CD54^{+}SDC2^{+} myogenic progenitors. Cells were counted every 3 days. Data are shown as the mean ± SEM of three independent biological replicates. $^{*}p < 0.05$. ns, not significant.
(H) Bar graphs show the percentage distribution of LV and GSH myogenic progenitors in different stages of the cell cycle. Data are shown as the mean ± SEM of three independent biological replicates. $^{*}p < 0.05$. ns, not significant.

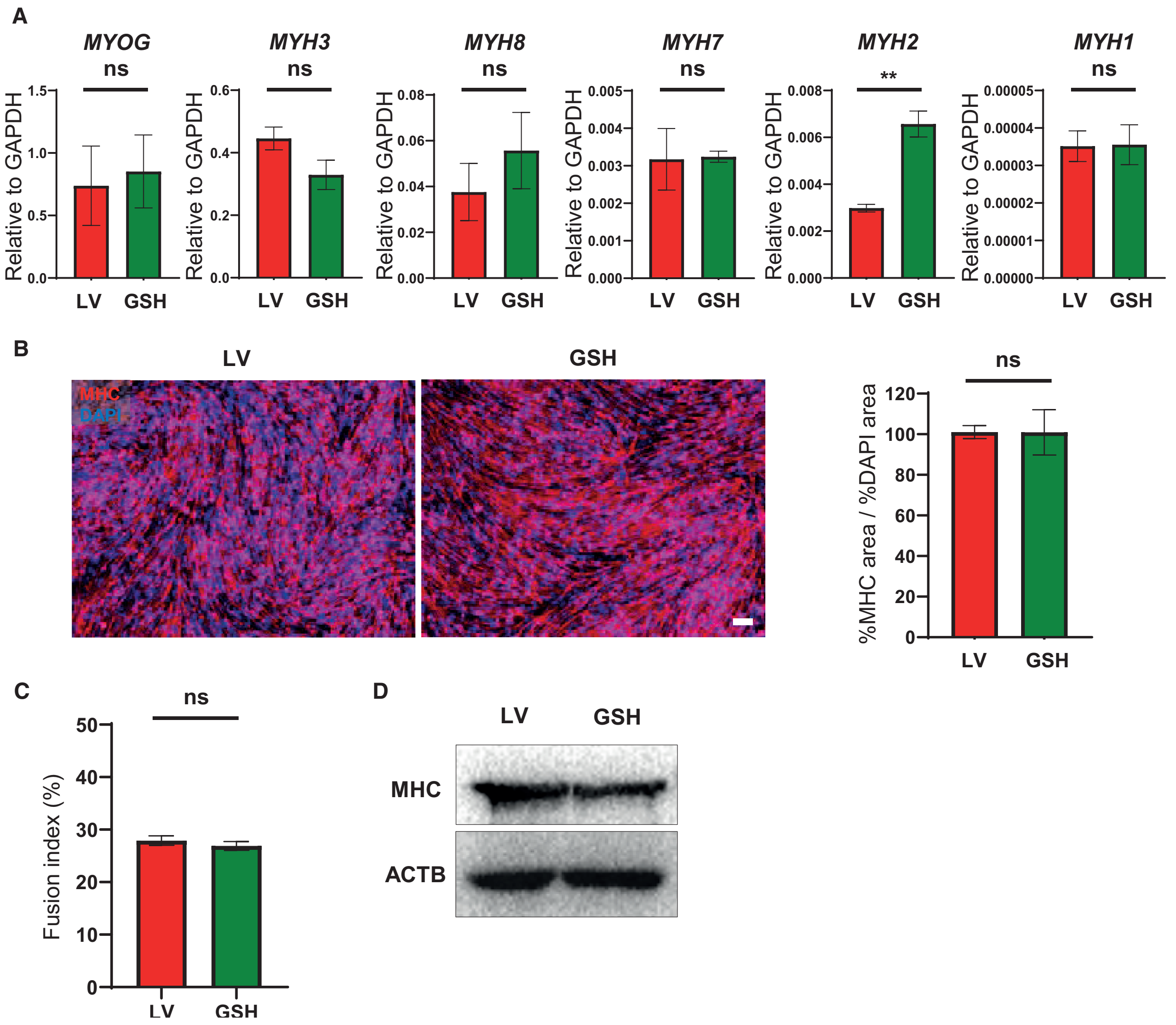

Figure 3. Terminal Differentiation of GSH Myogenic Progenitors into Myotubes

(A) Bar graphs show gene expression of *MYOGENIN* (*MYOG*) and *MYOSIN-HEAVY-CHAIN* (*MYH*) isoforms in GSH and LV iPSC-derived myotubes. Data are shown as the mean ± SEM of six independent replicates. **p < 0.01. ns, not significant.

(B) Representative images show immunofluorescence staining for MHC (red) in GSH and LV myotubes (left panels). DAPI (blue) stains nuclei. Scale bar, 100 μm. Right panel shows the ratio of percent MHC-stained area to percent DAPI area. Data are shown as the mean ± SEM of three independent replicates. ns, not significant.

(C) Fusion index quantification of LV and GSH myotubes. Data are shown as the mean ± SEM of three independent replicates. ns, not significant.

(D) Western blot for MHC. β-Actin was used as a loading control.

a positive correlation between PAX7 expression and proliferation rate. In any case, since GSH-generated myogenic progenitors double approximately every 2 days (47.2 ± 1.6 h), it should be feasible to obtain large numbers of GSH PAX7$^+$ myogenic progenitors for transplantation.

Taken together, our findings demonstrate feasibility for the use of GSH sites as an alternative approach to generate therapeutically relevant PSC-derived PAX7$^+$ myogenic progenitors.

EXPERIMENTAL PROCEDURES

Generation of PAX7$^+$rtTA$^+$ GSH PSC Lines

hROSA26 gRNA/Cas9n and AAVS1 ZFN expression plasmids, as well as pR26_CAG-rtTA and pAAVS1_TRE targeting plasmids, were kindly provided by Dr. Mark Kotter from the University Cambridge, UK (Pawlowski et al., 2017). We used SpeI/EcoRI sites to clone *PAX7* (Darabi et al., 2012) into the pAAVS1_TRE targeting

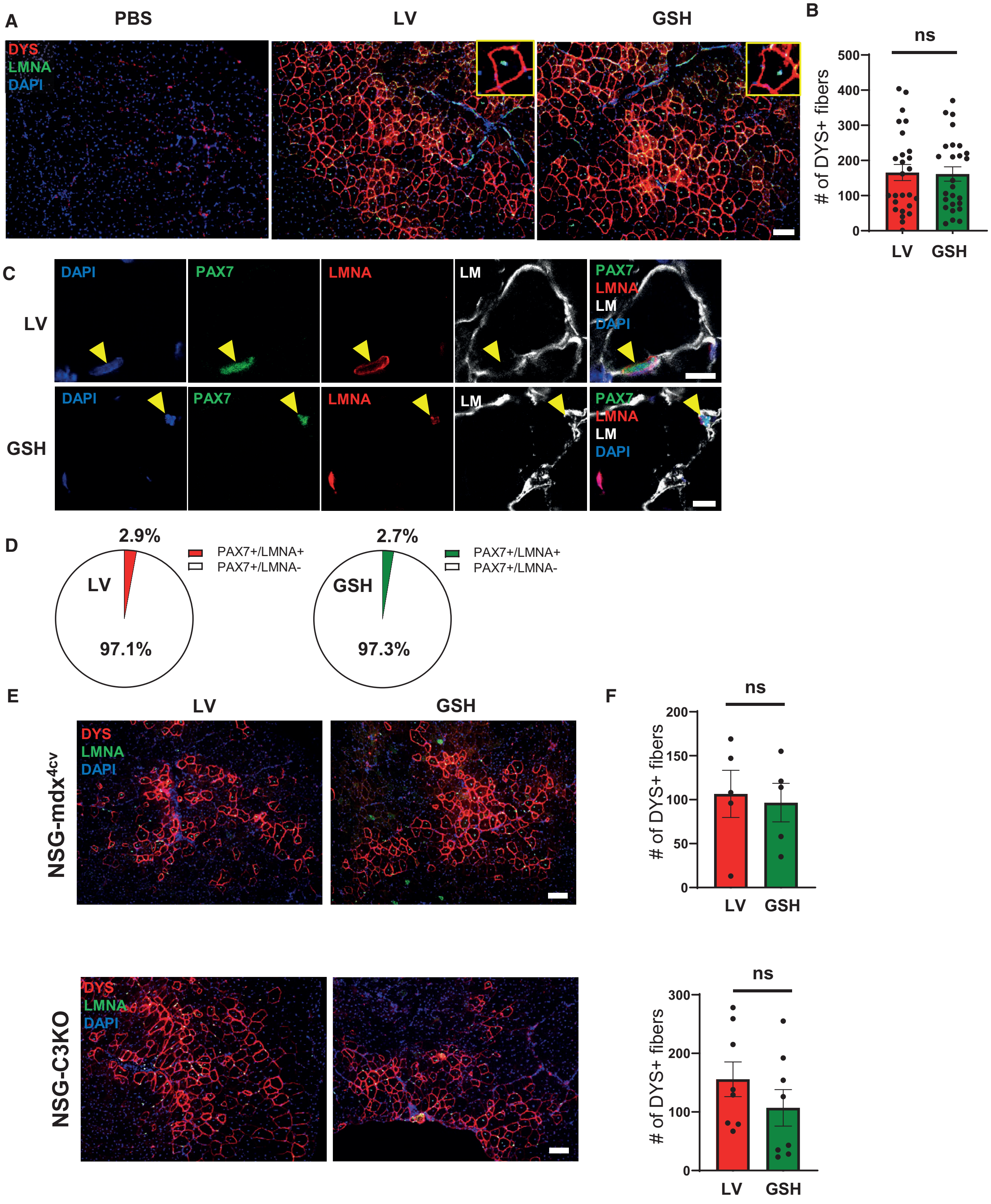

(legend on next page)

plasmid. hROSA26 gRNA/Cas9n expression plasmids and pR26_CAG-rtTA targeting plasmid were delivered to hPSCs by nucleofection (Lonza), as previously described (Pawlowski et al., 2017). The detailed description is provided in Supplemental Experimental Procedures.

hPSC Maintenance and Myogenic Differentiation

PSCs were maintained in mTeSR1 medium (STEMCELL Technologies) on Matrigel-coated plates. Myogenic differentiation was performed as previously described (Selvaraj et al., 2019b). The detailed description is provided in Supplemental Experimental Procedures.

FACS Analysis, Cell Cycle, and Sorting

A detailed description is provided in Supplemental Experimental Procedures.

Transplantation Studies

Animal experiments were carried out according to protocols approved by the University of Minnesota Institutional Animal Care and Use Committee. TA muscles of 6- to 8-week-old NSG (Jackson), and 10- to 12-week-old NSG-mdx^{4cv} and NSG-C3KO mice were pre-injured with cardiotoxin and injected with 1 million myogenic progenitors. The detailed description is provided in Supplemental Experimental Procedures.

qRT-PCR

A detailed description is provided in Supplemental Experimental Procedures.

Immunofluorescence Staining and Western Blot Analysis

A detailed description is provided in Supplemental Experimental Procedures.

Southern Blot Analysis

A detailed description is provided in Supplemental Experimental Procedures.

Statistical Analysis

Differences between the two samples were assessed by using the Student's t test for independent samples. p values lower than 0.05 were considered significant. Statistical analyses were performed using Prism 7 software (GraphPad).

SUPPLEMENTAL INFORMATION

Supplemental Information can be found online at https://doi.org/10.1016/j.stemcr.2020.11.001.

AUTHOR CONTRIBUTIONS

H.K. designed and performed experiments, analyzed the data, and wrote the manuscript; S.S., J.K., K.A., and B.I.G. performed experiments and analyzed the data; R.C.R.P. contributed to experimental design and interpretation of the data, and wrote the manuscript.

ACKNOWLEDGMENTS

We acknowledge the generous support from ADVault and MyDirectives.com (R.C.R.P.). This project was also supported by funds from the NIH, grants R01 AR055299 and AR071439 (R.C.R.P.). We thank Mark Kotter (University of Cambridge, UK) for targeting and expression plasmids. The monoclonal antibody to MHC was obtained from the Developmental Studies Hybridoma Bank developed under the auspices of the NICHD and maintained by the University of Iowa.

Received: April 28, 2020
Revised: November 1, 2020
Accepted: November 2, 2020
Published: December 3, 2020

REFERENCES

Albini, S., Coutinho, P., Malecova, B., Giordani, L., Savchenko, A., Forcales, S.V., and Puri, P.L. (2013). Epigenetic reprogramming of human embryonic stem cells into skeletal muscle cells and generation of contractile myospheres. Cell Rep. 3, 661–670.

Figure 4. *In Vivo* Regenerative Potential of GSH Myogenic Progenitors

(A) Representative images show immunofluorescence staining for DYSTROPHIN (DYS, red) and LAMIN A/C (LMNA, green) in TA muscles from NSG mice that had been transplanted with GSH and LV myogenic progenitors. PBS-injected muscles served as negative control (left panel). DAPI stains nuclei (blue). Scale bar, 100 μm.

(B) Graph shows quantification of engraftment (from A) as shown by the number of donor-derived human DYS$^+$ myofibers per section from TA muscles of 26 mice. Error bars represent mean ± SEM. ns, not significant.

(C) Satellite cell engraftment in transplanted muscles. Representative images show immunofluorescence staining for PAX7 (red), LMNA (green), and LAMININ (LM, gray). DAPI in blue stains nuclei. Scale bar, 10 μm.

(D) Percentage of PAX7$^+$/LMNA$^+$ (donor) cells and PAX7$^+$/LMNA$^-$ (recipient) cells per muscle section. Data are shown as mean ± SEM (n = 7 mice per group).

(E) Myofiber engraftment in dystrophic mouse models. Representative images show immunofluorescence staining for DYS (red) and LMNA (green) in transplanted TA muscles from NSG-mdx^{4cv} (upper panels) and NSG-C3KO (lower panels) mice. DAPI stains nuclei. Scale bar, 100 μm.

(F) Bar graph shows quantification of engraftment as measured by the maximum number of donor-derived human DYS$^+$ myofibers per section from TA muscles of NSG-mdx^{4cv} (n = 5 mice per group) and NSG-C3KO (n = 8 mice per group). Error bars represent mean ± SEM. ns, not significant.

Arpke, R.W., Darabi, R., Mader, T.L., Zhang, Y., Toyama, A., Lonetree, C.L., Nash, N., Lowe, D.A., Perlingeiro, R.C., and Kyba, M. (2013). A new immuno-, dystrophin-deficient model, the NSG-mdx(4Cv) mouse, provides evidence for functional improvement following allogeneic satellite cell transplantation. Stem Cells *31*, 1611–1620.

Azzag, K., Ortiz-Cordero, C., Oliveira, N.A.J., Magli, A., Selvaraj, S., Tungtur, S., Upchurch, W., Iaizzo, P.A., Lu, Q.L., and Perlingeiro, R.C.R. (2020). Efficient engraftment of pluripotent stem cell-derived myogenic progenitors in a novel immunodeficient mouse model of limb girdle muscular dystrophy 2I. Skelet. Muscle *10*, 10.

Barberi, T., Bradbury, M., Dincer, Z., Panagiotakos, G., Socci, N.D., and Studer, L. (2007). Derivation of engraftable skeletal myoblasts from human embryonic stem cells. Nat. Med. *13*, 642–648.

Baron, U., and Bujard, H. (2000). Tet repressor-based system for regulated gene expression in eukaryotic cells: principles and advances. Methods Enzymol. *327*, 401–421.

Bowles, D.E., McPhee, S.W., Li, C., Gray, S.J., Samulski, J.J., Camp, A.S., Li, J., Wang, B., Monahan, P.E., Rabinowitz, J.E., et al. (2012). Phase 1 gene therapy for Duchenne muscular dystrophy using a translational optimized AAV vector. Mol. Ther. *20*, 443–455.

Chal, J., Al Tanoury, Z., Hestin, M., Gobert, B., Aivio, S., Hick, A., Cherrier, T., Nesmith, A.P., Parker, K.K., and Pourquie, O. (2016). Generation of human muscle fibers and satellite-like cells from human pluripotent stem cells in vitro. Nat. Protoc. *11*, 1833–1850.

Collins, C.A., Gnocchi, V.F., White, R.B., Boldrin, L., Perez-Ruiz, A., Relaix, F., Morgan, J.E., and Zammit, P.S. (2009). Integrated functions of Pax3 and Pax7 in the regulation of proliferation, cell size and myogenic differentiation. PLoS One *4*, e4475.

Crudele, J.M., and Chamberlain, J.S. (2019). AAV-based gene therapies for the muscular dystrophies. Hum. Mol. Genet. *28*, R102–R107.

Darabi, R., Santos, F.N., Filareto, A., Pan, W., Koene, R., Rudnicki, M.A., Kyba, M., and Perlingeiro, R.C. (2011). Assessment of the myogenic stem cell compartment following transplantation of Pax3/Pax7-induced embryonic stem cell-derived progenitors. Stem Cells *29*, 777–790.

Darabi, R., Arpke, R.W., Irion, S., Dimos, J.T., Grskovic, M., Kyba, M., and Perlingeiro, R.C. (2012). Human ES- and iPS-derived myogenic progenitors restore DYSTROPHIN and improve contractility upon transplantation in dystrophic mice. Cell Stem Cell *10*, 610–619.

De Ravin, S.S., Reik, A., Liu, P.Q., Li, L., Wu, X., Su, L., Raley, C., Theobald, N., Choi, U., Song, A.H., et al. (2016). Targeted gene addition in human CD34(+) hematopoietic cells for correction of X-linked chronic granulomatous disease. Nat. Biotechnol. *34*, 424–429.

Emery, A.E.H. (2002). The muscular dystrophies. Lancet *359*, 687–695.

Goswami, R., Subramanian, G., Silayeva, L., Newkirk, I., Doctor, D., Chawla, K., Chattopadhyay, S., Chandra, D., Chilukuri, N., and Betapudi, V. (2019). Gene therapy leaves a vicious cycle. Front. Oncol. *9*, 297.

Goudenege, S., Lebel, C., Huot, N.B., Dufour, C., Fujii, I., Gekas, J., Rousseau, J., and Tremblay, J.P. (2012). Myoblasts derived from normal hESCs and dystrophic hiPSCs efficiently fuse with existing muscle fibers following transplantation. Mol. Ther. *20*, 2153–2167.

Hicks, M.R., Hiserodt, J., Paras, K., Fujiwara, W., Eskin, A., Jan, M., Xi, H., Young, C.S., Evseenko, D., Nelson, S.F., et al. (2018). ERBB3 and NGFR mark a distinct skeletal muscle progenitor cell in human development and hPSCs. Nat. Cell Biol. *20*, 46–57.

Incitti, T., Magli, A., Darabi, R., Yuan, C., Lin, K., Arpke, R.W., Azzag, K., Yamamoto, A., Stewart, R., Thomson, J.A., et al. (2019). Pluripotent stem cell-derived myogenic progenitors remodel their molecular signature upon in vivo engraftment. Proc. Natl. Acad. Sci. U S A *116*, 4346–4351.

Kim, J., Magli, A., Chan, S.S.K., Oliveira, V.K.P., Wu, J., Darabi, R., Kyba, M., and Perlingeiro, R.C.R. (2017a). Expansion and purification are critical for the therapeutic application of pluripotent stem cell-derived myogenic progenitors. Stem Cell Reports *9*, 12–22.

Kim, J., Oliveira, V.K.P., Yamamoto, A., and Perlingeiro, R.C.R. (2017b). Generation of skeletal myogenic progenitors from human pluripotent stem cells using non-viral delivery of minicircle DNA. Stem Cell Res. *23*, 87–94.

Lyu, C., Shen, J., Wang, R., Gu, H., Zhang, J., Xue, F., Liu, X., Liu, W., Fu, R., Zhang, L., et al. (2018). Targeted genome engineering in human induced pluripotent stem cells from patients with hemophilia B using the CRISPR-Cas9 system. Stem Cell Res. Ther. *9*, 92.

Magli, A., Incitti, T., Kiley, J., Swanson, S.A., Darabi, R., Rinaldi, F., Selvaraj, S., Yamamoto, A., Tolar, J., Yuan, C., et al. (2017). PAX7 targets, CD54, integrin alpha9beta1, and SDC2, allow isolation of human ESC/iPSC-derived myogenic progenitors. Cell Rep. *19*, 2867–2877.

Mendell, J.R., Kissel, J.T., Amato, A.A., King, W., Signore, L., Prior, T.W., Sahenk, Z., Benson, S., McAndrew, P.E., Rice, R., et al. (1995). Myoblast transfer in the treatment of Duchenne's muscular dystrophy. N. Engl. J. Med. *333*, 832–838.

Mendell, J.R., Campbell, K., Rodino-Klapac, L., Sahenk, Z., Shilling, C., Lewis, S., Bowles, D., Gray, S., Li, C., Galloway, G., et al. (2010). Dystrophin immunity in Duchenne's muscular dystrophy. N. Engl. J. Med. *363*, 1429–1437.

Montarras, D., Morgan, J., Collins, C., Relaix, F., Zaffran, S., Cumano, A., Partridge, T., and Buckingham, M. (2005). Direct isolation of satellite cells for skeletal muscle regeneration. Science *309*, 2064–2067.

Papapetrou, E.P., and Schambach, A. (2016). Gene insertion into genomic safe harbors for human gene therapy. Mol. Ther. *24*, 678–684.

Pawlowski, M., Ortmann, D., Bertero, A., Tavares, J.M., Pedersen, R.A., Vallier, L., and Kotter, M.R.N. (2017). Inducible and deterministic forward programming of human pluripotent stem cells into neurons, skeletal myocytes, and oligodendrocytes. Stem Cell Reports *8*, 803–812.

Selvaraj, S., Dhoke, N.R., Kiley, J., Mateos-Aierdi, A.J., Tungtur, S., Mondragon-Gonzalez, R., Killeen, G., Oliveira, V.K.P., Lopez de Munain, A., and Perlingeiro, R.C.R. (2019a). Gene correction of LGMD2A patient-specific iPSCs for the development of targeted autologous cell therapy. Mol. Ther. *27*, 2147–2157.

Selvaraj, S., Mondragon-Gonzalez, R., Xu, B., Magli, A., Kim, H., Laine, J., Kiley, J., McKee, H., Rinaldi, F., Aho, J., et al. (2019b). Screening identifies small molecules that enhance the maturation of human pluripotent stem cell-derived myotubes. eLife *8*, e47970.

Shelton, M., Metz, J., Liu, J., Carpenedo, R.L., Demers, S.P., Stanford, W.L., and Skerjanc, I.S. (2014). Derivation and expansion of PAX7-positive muscle progenitors from human and mouse embryonic stem cells. Stem Cell Reports *3*, 516–529.

Smith, J.R., Maguire, S., Davis, L.A., Alexander, M., Yang, F., Chandran, S., ffrench-Constant, C., and Pedersen, R.A. (2008). Robust, persistent transgene expression in human embryonic stem cells is achieved with AAVS1-targeted integration. Stem Cells *26*, 496–504.

Tedesco, F.S., Gerli, M.F.M., Perani, L., Benedetti, S., Ungaro, F., Cassano, M., Antonini, S., Tagliafico, E., Artusi, V., Longa, E., et al. (2012). Transplantation of genetically corrected human iPSC-derived progenitors in mice with limb-girdle muscular dystrophy. Sci. Transl. Med. *4*, 140ra189.

Wu, J., Matthias, N., Lo, J., Ortiz-Vitali, J.L., Shieh, A.W., Wang, S.H., and Darabi, R. (2018). A myogenic double-reporter human pluripotent stem cell line allows prospective isolation of skeletal muscle progenitors. Cell Rep. *25*, 1966–1981.e4.

Xi, H., Fujiwara, W., Gonzalez, K., Jan, M., Liebscher, S., Van Handel, B., Schenke-Layland, K., and Pyle, A.D. (2017). In vivo human somitogenesis guides somite development from hPSCs. Cell Rep. *18*, 1573–1585.

Young, C.S., Hicks, M.R., Ermolova, N.V., Nakano, H., Jan, M., Younesi, S., Karumbayaram, S., Kumagai-Cresse, C., Wang, D., Zack, J.A., et al. (2016). A single CRISPR-Cas9 deletion strategy that targets the majority of DMD patients restores dystrophin function in hiPSC-derived muscle cells. Cell Stem Cell *18*, 533–540.

Zou, J., Sweeney, C.L., Chou, B.K., Choi, U., Pan, J., Wang, H., Dowey, S.N., Cheng, L., and Malech, H.L. (2011). Oxidase-deficient neutrophils from X-linked chronic granulomatous disease iPS cells: functional correction by zinc finger nuclease-mediated safe harbor targeting. Blood *117*, 5561–5572.

Stem Cell Reports, Volume *16*

Supplemental Information

Genomic Safe Harbor Expression of PAX7 for the Generation of Engraftable Myogenic Progenitors

Hyunkee Kim, Sridhar Selvaraj, James Kiley, Karim Azzag, Bayardo I. Garay, and Rita C.R. Perlingeiro

Supplemental Figure 1

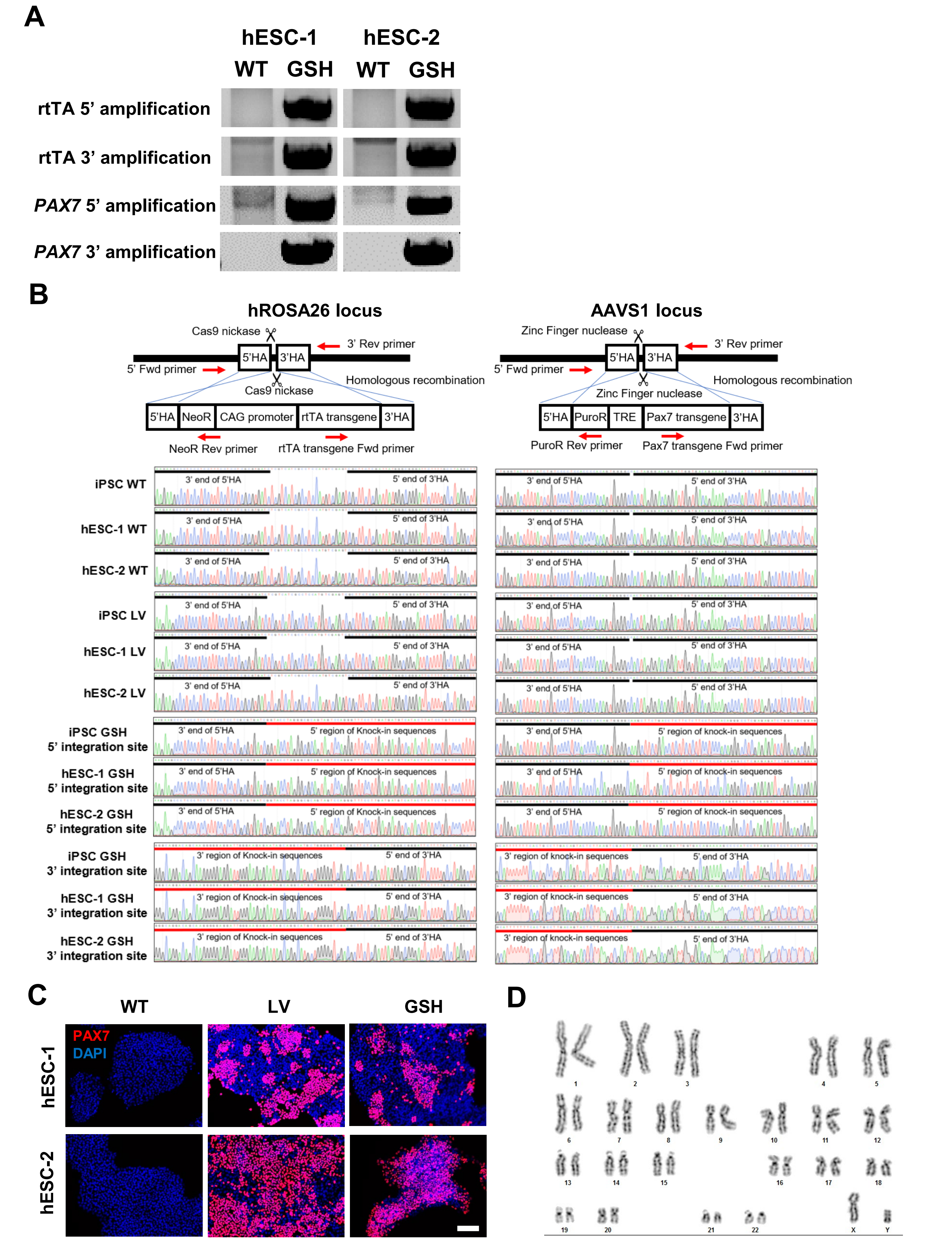

Supplemental Figure 2

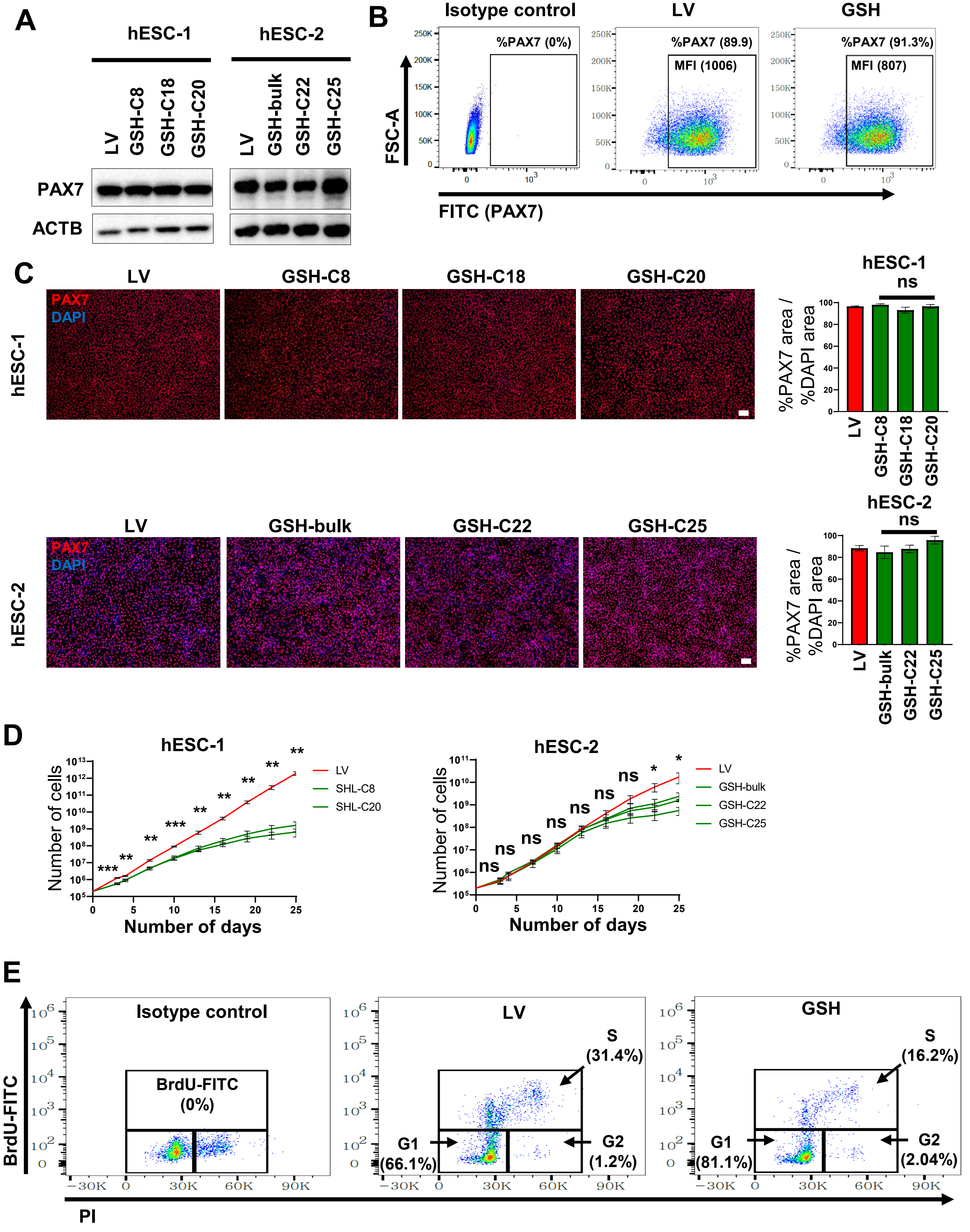

Supplemental Figure 3

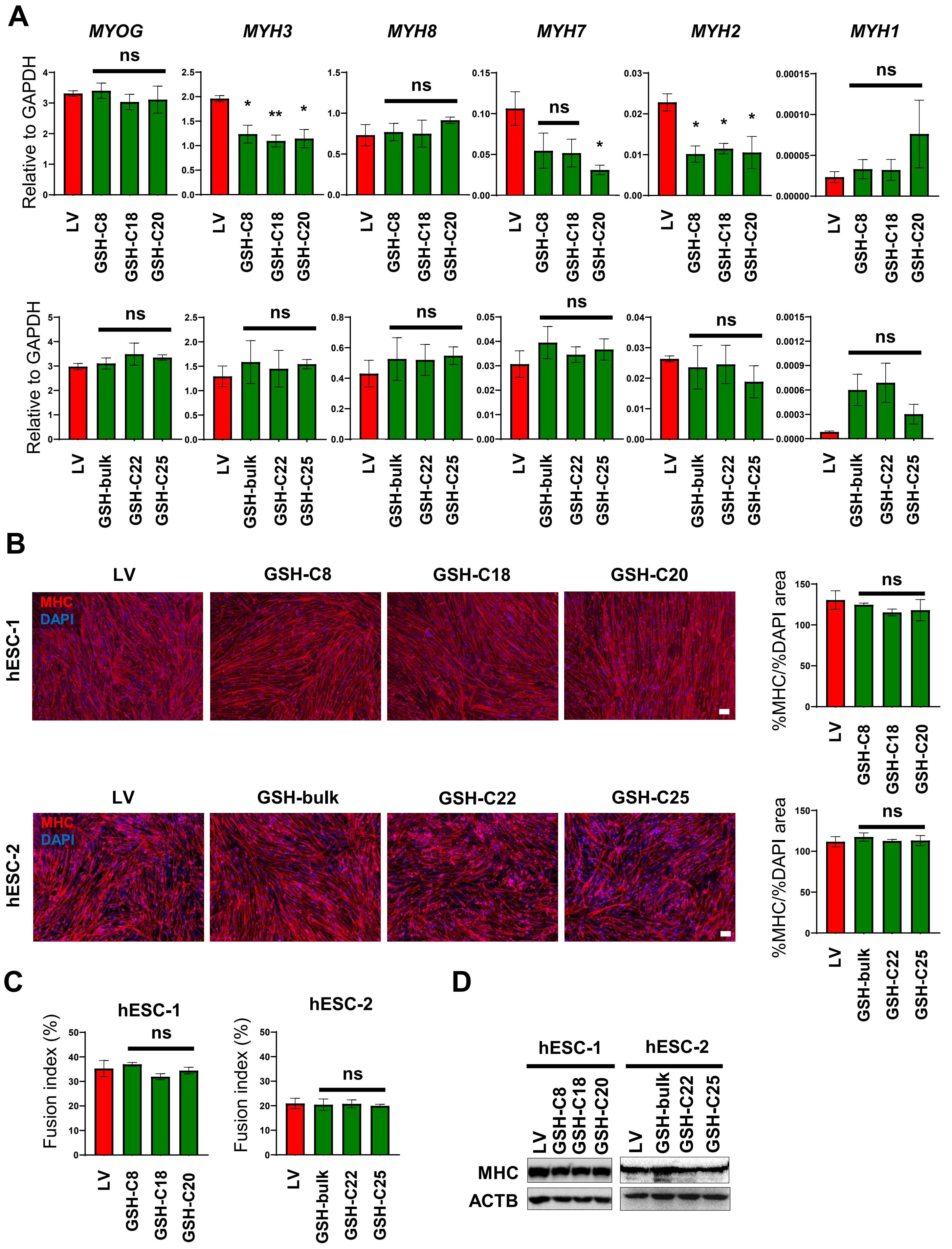

SUPPLEMENTAL FIGURE LEGENDS

Figure S1. Related to Figure 1: Confirmation of *PAX7* and rtTA insertion into respective AAVS1 and ROSA26 loci in additional hPSC lines.
(A) PCR shows integration analysis of *PAX7* and rtTA transgenes into the AAVS1 and ROSA26 loci in hESC-1 and hESC-2 lines. Primers were designed outside of the 5'- and 3'-homology arms and within the transgenes. WT: wild-type parental hESC; GSH: genomic safe harbor targeted hESC.
(B) Sequencing results of the rtTA and *PAX7* transgene knock-in in the ROSA26 and AAVS1 loci. Primers amplifying knock-in specific sequences were used to obtain the amplicons for sequencing. Black lines indicate intact sequences whereas red lines indicate knock-in sequences.
(C) Immunofluorescence staining shows PAX7 protein expression upon 1 day of dox induction in hESC-1 and hESC-2 lines. Of note, this staining was performed in the bulk population prior to clonal selection. PAX7 staining is shown in red. DAPI in blue stains nuclei. WT and LV hESC-1/hESC-2 were used as negative and positive controls, respectively. Scale bar, 100 μm.
(D) Cytogenetic analyses show normal karyotype for representative GSH iPSC line (TC1133).

Figure S2. Related to Figure 2: Characterization of GSH PAX7$^+$ myogenic progenitors derived from the bulk and selected clones from targeted hESC-1 and hESC-2 lines.
(A) Western blot for PAX7. β-Actin was used as a loading control.
(B) Representative FACS plots for PAX7 in LV and GSH myogenic progenitors. Percentage for PAX7 and the mean fluorescence intensity (MFI) are indicated on respective plots. Isotype antibody served as negative control.
(C) Left panels show representative images of immunofluorescence staining for PAX7 (red) in GSH and LV CD54$^+$SDC2$^+$ myogenic progenitors derived from hESC-1 (upper panels) and hESC-2 (lower panels) lines. DAPI (blue) stains nuclei. Scale bar, 100 μm. Graph bars (right panels) show ratio of % PAX7-stained area to % DAPI area in GSH and LV myogenic progenitors. Data are shown as the mean ± S.E.M. of three independent replicates.
(D) Growth curves of GSH and LV CD54$^+$SDC2$^+$ myogenic progenitors derived from hESC-1 (left) and hESC-2 (right) lines. Cells were counted every 3 days. Data are shown as the mean ± S.E.M. of three independent replicates. *$p<0.05$, **$p<0.01$, ***$p<0.001$
(E) Representative FACS plots for cell cycle analysis. Isotype antibody served as negative control.

Figure S3. Related to Figure 3: Terminal differentiation of GSH hESC-derived myogenic progenitors into myotubes.
(A) Graph bars show gene expression of *MYOGENIN (MYOG)* and *MYOSIN-HEAVY-CHAIN* (*MYH*) isoforms in GSH and LV hESC-1 (upper) and hESC-2 (lower) myotubes. Data are shown as the mean ± S.E.M. of three independent replicates. *$p<0.05$, **$p<0.01$
(B) Left panels show representative images of immunofluorescence staining for MHC (red) in GSH and LV hESC-1 (upper) and hESC-2 (lower) myotubes. DAPI (blue) stains nuclei. Scale bar, 100 μm. Graph bar (right panel) shows ratio of % MHC-stained area to % DAPI area in GSH and LV myotubes derived from hESC-1 and hESC-2 lines. Data are shown as the mean ± S.E.M. of three independent replicates.
(C) Bar graph shows the quantification of fusion index of hESC-derived LV and GSH myotubes. Data are shown as the mean ± S.E.M. of three independent replicates.
(D) Western blot for MHC. β-Actin was used as a loading control.

SUPPLEMENTAL TABLES

Table S1. Primers for genotyping PCR

Locus	PCR types	Binding site of primers	Sequences
AAVS1	5' region integration	Endogenous genome (5')-Forward	TAGCCACTCTGTGCTGACCACTCTG
		PuroR (Vector)- Reverse	GAAGAGTTCTTGCAGCTCGGTGAC
	3' region integration	PAX7 (Vector)- Forward	CTGTCTCCTCAGGTGATGAGCATCTTG
		Endogenous genome (3')- Reverse	ACTGAGGGTTTCAGTGCTAAAACTAGGCTGT
	Locus PCR	Endogenous genome (5')- Forward	TAGCCACTCTGTGCTGACCACTCTG
		Endogenous genome (3')- Forward	AGCTCTTCTCTGTTCAGCCCTAAGAATC
hROSA26	5' region integration	Endogenous genome (5')- Forward	ACATAATGTTTGTTACGTTGGGAGGGAAAG
		NeoR (Vector)- Reverse	ATTGCATCAGCCATGATGGATACTT
	3' region integration	rtTA (Vector)- Forward	GAAACTCGCTCAAAAGCTGGG
		Endogenous genome (3')- Reverse	ACAGTACAAGCCAGTAATGGAG
	Locus PCR	Endogenous genome (5')- Forward	GTCTCTTTTCTGTCGGACCCTTACCTTGTC
		Endogenous genome (3')- Forward	ATCAGAGTATACTAGGAGCTCAGGAGTACAAGA

Table S2. SYBR green qRT-PCR primers

Target	Primers for SYBR green	Primer sequences
Endogenous PAX7	5'-UTR forward primer	TGTGTGTGGAGGGGAGGGAGAA
	PAX7 Exon2 reverse primer	GCTGATTGACCCGGCCTTGG

Table S3. Southern blot primers for probes

Southern blot probes	Primers for probe	Primer sequences
rtTA probe	rtTA forward primer	CCGCTGTGCTCTCCTCTCACATCG
	rtTA reverse primer	TCAGAAGTGGGGGCATAGAATCGG
PAX7 probe	PAX7 forward primer	CAAGTTCGGGAAGAAAGAGGA
	PAX7 reverse primer	GTATATGTCTGGGTAGTGGGTCCT

SUPPLEMENTAL EXPERIMENTAL PROCEDURES

Generation of PAX7^{+}rtTA^{+} GSH PSC lines.

hROSA26 gRNA/Cas9n and AAVS1 ZFN expression plasmids, as well as pR26_CAG-rtTA and pAAVS1_TRE targeting plasmids were kindly provided by Dr. Mark Kotter from the University Cambridge, UK (Pawlowski et al., 2017). We used SpeI/EcoRI sites to clone PAX7 (Darabi et al., 2012) into the pAAVS1_TRE targeting plasmid. hROSA26 gRNA/Cas9n expression plasmids and pR26_CAG-rtTA targeting plasmid were delivered to hPSCs by nucleofection (Lonza), as previously described (Pawlowski et al., 2017). Following G418 treatment (50 µg/mL for 10 days), neomycin resistant cells were selected. Subsequently, AAVS1 ZFN expression plasmids and pAAVS1_TRE-PAX7 targeting plasmid were delivered through nucleofection using the same procedure. Following puromycin treatment (500 ng/mL) for 10 days, resistant clones were selected and verified by knock-in specific PCR, sequencing, and protein expression. Sequences of PCR primers can be found in Table S1.

hPSC maintenance and myogenic differentiation

The following PSC lines were used: TC1133 iPS cells, and human ES cell lines H1 (hESC-1) and H9 (hESC-2). Cells were maintained in mTeSR1 medium (Stem Cell Technologies) on Matrigel-coated plates. PSCs were dissociated with Accutase (Innovative Cell Technologies) and passaged once they reached 90% confluency. For differentiation, PSCs (LV and GSH) were dissociated into single cells using Accutase, and differentiated into embryoid bodies (EBs) in the presence of EB myogenic medium, which consists of IMDM containing 4% fetal bovine serum, 11% KnockOut Serum Replacement (Gibco), 1% insulin-transferrin selenium (Gibco), 1% penicillin/streptomycin (Gibco), 1% Glutamax (Gibco), and 50 µg/mL ascorbic acid (Sigma). Doxycycline (dox; Sigma-Aldrich) induction (1 µg/ml) started on day 5 of EB differentiation. On day 8, medium was further supplemented with 5 ng/ml of human basic fibroblast growth factor (bFGF; PeproTech). On day 12, EBs were dissociated with 0.25% trypsin-EDTA, and plated as monolayer. On day 15, monolayer cultures were rinsed with PBS, and dissociated with Cellstripper (Corning) for fluorescence-activated cell sorting (FACS), as described below. CD54+SDC2 sorted cells were expanded in the EB myogenic medium described above with 1 µg/mL dox (Sigma) and 5ng/mL human basic FGF (PeproTech). For myotube differentiation, we used >95% confluent myogenic progenitors. These were rinsed with PBS to remove dox, and cultured in low serum conditions, as described previously (Darabi et al., 2012).

FACS analysis and sorting

To purify myogenic progenitors, cells were first stained with the primary antibodies biotin-conjugated CD54 (Thermo Fisher) and APC-conjugated SYNDECAN2 (SDC2; R&D systems) for 20 min, as previously described (Magli et al., 2017). Streptavidin PE-Cy7 (Thermo Fisher) was used for secondary staining. Cells were washed with PBS and then resuspended in PBS supplemented with 10% FBS. For PAX7 staining, cells were dissociated with 0.25% trypsin-EDTA, fixed with 4% PFA at room temperature for 10 min, and washed twice with PBS. Cell permeabilization was performed using FACS buffer containing 0.2% Tween, 0.5% Saponin, 4% FBS, and 0.4% 0.5M EDTA on a shaker for 30 min at room temperature. Next, cells were incubated with the PAX7 antibody (mouse; PAX7-s; DSHB) in FACS buffer without 0.2% Tween 20 at room temperature for 45 min. Cells were washed twice with PBS and incubated with an anti-mouse secondary antibody (anti-mouse Alexa Fluor 488) in FACS buffer without 0.2% Tween 20 for 45min. Cells were washed twice with PBS, and resuspended in FACS buffer containing 4% FBS, and 0.4% 0.5M EDTA. Samples were analyzed and sorted using a FACSAria II (BD Biosciences) and data were analyzed with FlowJo software.

Transplantation studies

Animal experiments were carried out according to protocols approved by the University of Minnesota Institutional Animal Care and Use Committee. Both TA muscles of 6-8 week-old NOD-scid IL2Rgnull (NSG; Jackson) or 10-12 week-old NOD-scid IL2Rgnull mdx4cv (NSG-mdx4cv) (Arpke et al., 2013) or 10-12 week-old NOD-scid IL2Rgnull C3KO (NSG-C3KO) (Selvaraj et al., 2019a) mice were pre-injured with 15 µl of cardiotoxin 10 µM (Latoxan). One day after cardiotoxin, TA muscles were injected with 10^6 myogenic progenitors. GSH myogenic progenitors were injected in one TA while the contralateral TA received LV myogenic progenitors, as internal control. CTX-injured TA muscles injected with PBS served as negative. TA muscles were collected for immunofluorescence analysis 8-9 weeks later.

Quantitative RT-PCR (qPCR)

Cells were lysed using Trizol reagent (Thermo Fisher) and RNA was extracted using purelink RNA mini kit (Thermo Fisher) with on-column DNAse treatment following manufacturer's instructions. RNA concentration was quantified using Nanodrop. For quantitative RT-PCR analysis, reverse transcription was performed using Superscript Vilo cDNA synthesis kit (Thermo Fisher) as per manufacturer's instruction. qPCR was performed using taqman probes (Applied Biosystems). For each qPCR reaction in 384-well plate, cDNA amount corresponding to 10 ng of total RNA, 0.5 µl of taqman probe or 0.2 µM of forward and reverse SYBR green primers and 5 µl of 2X master mix was utilized. qPCR was performed using QuantStudio 6 Flex Real-Time PCR System and the C_t values were determined. C_t value for gene of interest was normalized to that of the housekeeping control using the 2^-delta C_t calculation and values compared between the GSH and LV groups. Following are the taqman probes used in this study, *T* (Hs01084475_g1), *FOXC2* (Hs00270951_s1), *MEOX1* (Hs00244943_m1), *TCF15* (Hs00231821_m1), *PAX3* (Hs00992437_m1), *PAX7* (Hs00242962_m1), *PAX7* variant2 (Hs01557428_mH), *MYH1* (Hs00428600_m1), *MYH2* (Hs00430042_m1), *MYH3* (Hs01074230_m1), *MYH7* (Hs01110632_m1), *MYH8* (Hs00267293_m1), *MYOG* (Hs01072232_m1), and *GAPDH* (Hs99999905_m1). SYBR green primers are listed in Table S2.

Immunofluorescence staining

Cells were fixed at room temperature with 4% PFA for 30 min, followed by 15 min permeabilization with 0.3% Triton X-100 in PBS. Samples were then blocked with 3% BSA in PBS for 1 hr. We used the following primary and secondary antibodies: PAX7 (mouse; PAX7-s; DSHB), MHC (mouse; MF-20-s; DSHB), human DYSTROPHIN (mouse; MANDYS106(2C6)-s; DSHB), human LAMIN A/C (rabbit; ab108595; Abcam), LAMININ α2 (rat; sc-59854; Santa Cruz), and fluorophore-conjugated secondary antibodies (anti-mouse Alexa Fluor 555 (A-21426), anti-rabbit Alexa Fluor 488 (A-11008), and anti-rat Alexa Fluor 647 (A-21247); Thermo Fisher). Samples were incubated in primary antibody diluted in 3% BSA at 4°C overnight. Next day, samples were washed with PBS three times and incubated with secondary antibody and DAPI at room temperature for 1 hr. After incubation, samples were washed with PBS three times and stored at 4°C in dark until imaging. For tissue, muscle samples were cryosectioned at 14 µm thickness on glass slides. Sectioned tissues were then rehydrated in PBS for 5 min, fixated with 4% PFA for 10 min, and stained as described above. After the final wash, slides were mounted with coverslips by using ProLong Gold Antifade Mountant with DAPI (Thermo Fisher), and stored at 4°C in the dark until imaging. Samples were imaged using upright (Zeiss) and confocal (NikonNiE C2) microscopy. Quantification was performed using the Fiji software.

Western blot analysis

Cells were lysed in lysis buffer containing 20 mM Tris-HCl (Sigma), 0.1 mM EDTA (Sigma), 1 mM DDT (Sigma), 28 µM E64 (Sigma), 20 µg/mL soybean trypsin inhibitor (Sigma), and 2 mM phenylmethylsulfonyl fluoride (PMSF; Santa Cruz), and 1X Laemmli sample buffer. Cell scraper was used to collect lysate and the lysate was

boiled at 95°C for 5-10 min. Total protein concentration in lysate was measured by using a Bradford assay. 7.5% SDS-PAGE gels were used for electrophoresis and migrated proteins were transferred to immobilon-FL polyvinylidene fluoride (PVDF) membrane (Millipore). Transferred membrane was blocked in 5% dry milk (RPI) in PBST (PBS and 0.1% Tween 20) for 1 hr at room temperature. Blocked membrane was then incubated with primary antibody diluted in 5% BSA in PBST and incubated at 4°C overnight. Next day, membrane was washed three times in PBST and incubated with horseradish peroxidase (HRP)-conjugated secondary antibody for 1 hr at room temperature. Membrane was then washed three times with PBST and then Supersignal West chemiluminescent substrate (Thermo Fisher) was applied to image in Bio-Rad ChemiDoc MP imaging system. Antibodies used include TetR (mouse; 631131; Clontech), PAX7 (mouse; PAX7-s; DSHB), MHC (mouse; MF-20-s; DSHB), ACTB (mouse; sc-47778; Santa Cruz), and TUBB (mouse; sc-5274; Santa Cruz).

Southern blot analysis

Genomic DNA was extracted using the Purelink ™ Genomic DNA mini kit as per manufacturer's instructions. Genomic DNA was digested overnight at 37°C with BstXI restriction enzymes for detection of the rtTA transgene detection, and with XmnI for detection of *PAX7*. Following electrophoresis in 1% agarose gel, DNA was transferred to positively charge nylon membrane and probed with digoxigenin (DIG)-labeled rtTA and *PAX7* probes. DIG-labeled probes were synthesized by using PCR DIG Probe Synthesis Kit (Roche) and detection was performed by using DIG-High Prime DNA Labeling and Detection Starter Kit II (Roche). Primer sequences utilized for generating probes are listed in Table S3.

Cell cycle

For assessment of cell cycle we used the previously reported protocol (Zhu, 2012). Briefly, cells were treated with 30 μM BrdU (Sigma) for 8 hr, and following trypsinization, fixed and permeabilized with 70% ice-cold EtOH overnight at 4°C. For DNA denaturation, we used 2 N HCl with 0.5% Triton-X-100. Cells were washed first with 0.1 M sodium tetraborate (pH 8.5) in 1X PBS solution, and then with 1% BSA in 1X PBS. Cells were incubated with a FITC-conjugated BrdU antibody (mouse; 364104; BioLegend) for 1hr at room temperature in the dark, washed with 1% BSA in 1X PBS, resuspended in 1X PBS containing 10 μg/mL RNase A and 20 μg/mL propidium iodide, and incubated at room temperature for 30 min in the dark. Samples were analyzed immediately or stored at 4°C in the dark until FACS analysis. FlowJo software was used for data analysis.

SUPPLEMENTAL REFERENCES

Zhu, H. (2012). Cell proliferation assay by flow cytometry (BrdU and PI staining). Bio-101 2, e198.
